# Fully Automated GMP-Compliant Synthesis of [^18^F]FE-PE2I

**DOI:** 10.3390/ph14070601

**Published:** 2021-06-22

**Authors:** Klas Bratteby, Charlotte Lund Denholt, Szabolcs Lehel, Ida Nymann Petersen, Jacob Madsen, Maria Erlandsson, Tomas Ohlsson, Matthias Manfred Herth, Nic Gillings

**Affiliations:** 1 Department of Drug Design and Pharmacology, University of Copenhagen, Jagtvej 160, 2100 Copenhagen, Denmark; klas.bratteby@sund.ku.dk (K.B.); matthias.herth@sund.ku.dk (M.M.H.); 2 Department of Clinical Physiology Nuclear Medicine PET, Copenhagen University Hospital Rigshospitalet, Blegdamsvej 9, 2100 Copenhagen, Denmark; charlotte.lund.denholt@regionh.dk (C.L.D.); szabolcs.lehel@regionh.dk (S.L.); ida.nymann.petersen@regionh.dk (I.N.P.); jacob.madsen@regionh.dk (J.M.); 3 Department of Radiation Physics, Skåne University Hospital, Barngatan 3, 22242 Lund, Sweden; maria.n.erlandsson@skane.se (M.E.); tomas.ohlsson@skane.se (T.O.)

**Keywords:** [^18^F]FE-PE2I, radiochemistry, fluorine-18, automation, GMP

## Abstract

In the struggle to understand and accurately diagnose Parkinson′s disease, radiopharmaceuticals and medical imaging techniques have played a major role. By being able to image and quantify the dopamine transporter density, noninvasive diagnostic imaging has become the gold standard. In the shift from the first generation of SPECT tracers, the fluorine-18-labeled tracer [^18^F]FE-PE2I has emerged as the agent of choice for many physicians. However, implementing suitable synthesis for the production of [^18^F]FE-PE2I has proved more challenging than expected. Through a thorough analysis of the relevant factors affecting the final radiochemical yield, we were able to implement high-yielding fully automated GMP-compliant synthesis of [^18^F]FE-PE2I on a Synthera^®^+ platform. By reaching RCYs up to 62%, it allowed us to isolate 25 GBq of the formulated product, and an optimized formulation resulted in the shelf life of 6 h, satisfying the increased demand for this radiopharmaceutical.

## 1. Introduction

Positron emission tomography (PET) is a medical imaging technique that can noninvasively study molecular processes in the brain and body without imparting physiological effects due to the use of tracer amounts of radiopharmaceuticals [1,2]. The radiolabeled dopamine transporter ligand, [^18^F]FE-PE2I, has over the past few years been shown to be a valuable tool for the study of patients with Parkinson′s disease [3,4,5]. Whilst there is an authorized single-photon emission computed tomography (SPECT) radiopharmaceutical available for such investigations, namely [^123^I]FP-CIT ([^123^I]ioflupane, DaTscan™) [6], PET scanning with [^18^F]FE-PE2I has several clear advantages such as reduced radiation burden and better discrimination between healthy patients and early-stage Parkinson′s disease patients [7,8].

**Figure 1:**
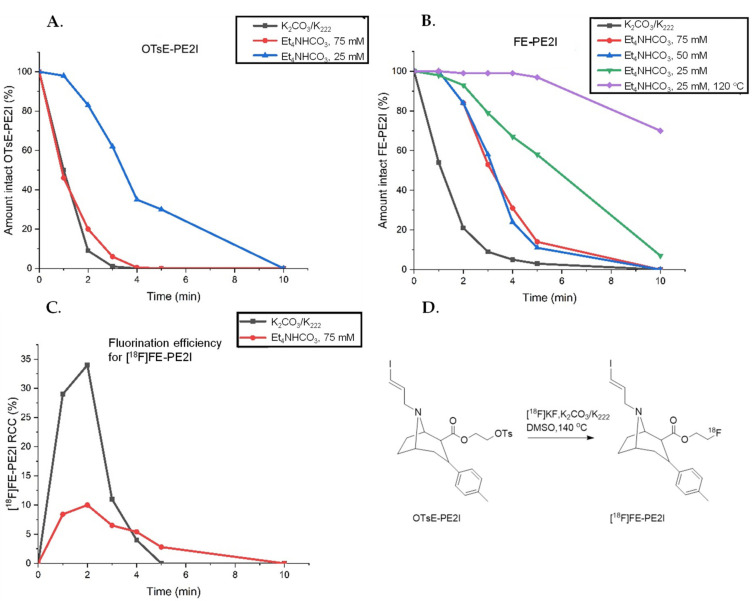
(**A**) Stability of the tosylate precursor (OTsE-PE2I) evaluated in DMSO at 140 °C added to the dried QMA/PS-30 eluate. (**B**) Stability of the reference FE-PE2I evaluated in DMSO at 140 °C (if nothing else is stated) added to the dried QMA/PS-30 eluate. (**C**) Radiochemical conversion for [^18^F]FE-PE2I using QMA/K_2_CO_3_/K_222_ or PS-30/Et_4_NHCO_3_ elution in DMSO at 140 °C over time. (**D**) Radiolabeling reaction to form [^18^F]FE-PE2I.

The synthesis of [^18^F]FE-PE2I was first reported in 2009 [9] and a subsequent publication provided a simpler one-step radiolabeling procedure (Figure 1D) [10]. The synthesis of [^18^F]FE-PE2I based on the published procedure was implemented and scaled up at Copenhagen University Hospital, Rigshospitalet, in 2017 and, following approval for human use by the Danish Medicines Agency (compassionate use permit), more than 300 instances of production have been performed to date. The initial production process utilized an automated radiochemistry system (Scansys Laboratorieteknik ApS) with a preparative Onyx C18 high-performance liquid chromatography (HPLC) system. Over time and with experience, the process was optimized and the amount of radioactivity that could be produced was increased. This process was almost fully automated; however, due to technical issues, the precursor solution was manually added to the reaction vial. Due to increasing demand and to minimize radiation exposure to the personnel, the process was transferred to an automated cassette-based radiosynthesis system (Synthera^®^+, IBA) with a preparative HPLC module. Furthermore, following an intensive investigation of conditions for elution of the [^18^F]fluoride-trapping cartridge, radiochemical yields (RCYs) were improved significantly [11]. Herein, we report data on the optimization of the synthesis, stabilization of the product along with a presentation of the validation of the production process and quality control according to the good manufacturing practice (GMP).

## 2. Results and Discussion

### 2.1. General Considerations for the Production of [^18^F]FE-PE2I

Before implementation, the reaction parameters for the one-step synthesis from the tosylate precursor were optimized with inspiration from the results published by Stepanov et al. in 2012 [10]. Elution of the QMA cartridge with 15.6 µmol Kryptofix (K_222_), 7.8 µmol potassium carbonate (K_2_CO_3_) and the reaction time of 2 min at 135 °C using 1 mg precursor in 600 µL DMSO was found to yield adequate results, with overall RCYs up to 15%.

**Figure 2:**
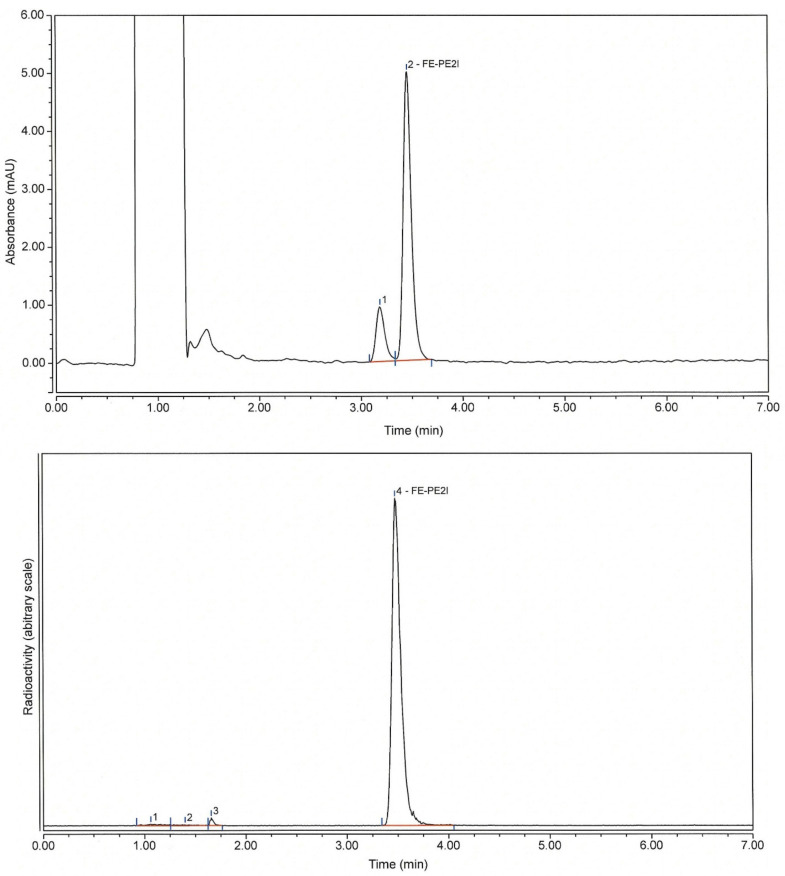
Analytical HPLC chromatograms of [^18^F]FE-PE2I (UV—220 nm) (**top**) and radioactivity (**bottom**).

Analytical methods: An HPLC method was developed in order to determine the radiochemical purity (RCP) along with the content of the active substance (FE-PE2I) and any impurities. With this method, determination of the amount of free [^18^F]fluoride was possible. As can be seen in Figure 2, along with [^18^F]fluoride, there were 2–3 small radioactive impurities in the product. The UV chromatogram revealed one related impurity, which had a similar UV spectrum, but was not identified. The HPLC method was validated according to the ICH guidelines [12]. A summary of validation parameters and results can be seen in Table 1. An existing GC method was used for determination of ethanol content along with any residual solvents (see Section 3.6.2.).

### 2.2. Production of [^18^F]FE-PE2I Using a Scansys Module

#### 2.2.1. Manual Optimization of Reaction Parameters

The reaction was optimized using 1 mg precursor per synthesis. The highest radiochemical conversion (RCC) [13] of [^18^F]FE-PE2I was reported to be reached using DMSO or DMF as a solvent at 140 °C for 5 min [10]. Using this as a starting point, the stability of the precursor and the reference compound (FE-PE2I) was analyzed using two different methods for elution of the trapped [^18^F]fluoride from the anion exchange cartridge. The previously published elution using K_2_CO_3_/K_222_ of a CO_3_^2−^-preconditioned QMA (Sep-Pak Light QMA, Waters) was compared with the less basic Et_4_NHCO_3_ elution of an HCO_3_^−^-preconditioned Chromafix PS-30 (IBA RadioPharma Solutions). The experiments were conducted by mimicking the reaction conditions but without adding [^18^F]fluoride (Figure 1A,B). Amounts of the precursor and FE-PE2I were determined using HPLC analysis. The results were compared with the RCC of [^18^F]FE-PE2I in manual reactions using the same conditions (Figure 1C,D).

The results show that both the precursor and the formed product degraded over time using the harsh K_2_CO_3_/K_222_ elution of a QMA cartridge. The milder Et_4_NHCO_3_ elution of the HCO_3_-preconditoined PS-30 cartridge slightly improved this, presumably due to the milder HCO_3_^−^ anions used for both preconditioning and elution. This was corroborated by manual ^18^F-fluorination experiments, where the product degraded completely after 5 min of the reaction using the K_2_CO_3_/K_222_ elution conditions. However, using the milder Et_4_NHCO_3_ elution proved to be very inefficient in the radiolabeling and the RCC never exceeded 10%. Thus, the original K_2_CO_3_/K_222_ elution was chosen to proceed with. The reaction time was shortened to 2 min and the reaction temperature was lowered to 135 °C.

#### 2.2.2. Preparative HPLC Method

In order to reduce the synthesis time and minimize the amount of purification steps, a preparative HPLC method was developed using a buffered ethanol eluent containing citrate as an antioxidant for radical scavenging, as the product showed signs of radiolysis in initial experiments. The preparative HPLC column chosen was a reversed-phase monolithic column (Onyx C18, 10 × 150 mm, Phenomenex) eluted with 96% ethanol/25 mM sodium citrate buffer, pH 4.0 (20/80), at a flow rate of 5.5 mL/min and the collected fraction was sterile-filtered and diluted in phosphate-buffered saline (100 mM, pH 7.4) for injection without further purification. This obviates the need for a C18 solid-phase extraction step to switch to the solvents compatible with the formulation that could cause increased radiolysis [14].

At the end of 2018, the preparative HPLC eluent was further developed to a sodium ascorbate/ascorbic acid buffer (25 mM, pH 4.3) with 18% ethanol in order to improve the stability of the product at higher levels of radioactivity (see Section 2.2.3 and Section 2.2.4 below).

#### 2.2.3. Automated Production of [^18^F]FE-PE2I Using a Scansys Module

The optimized reaction parameters and purification method were set up on a Scansys synthesis module. Unfortunately, the initial syntheses failed to furnish [^18^F]FE-PE2I in the expected yields and purity. It was discovered that this was due to water in the reaction mixture from the robotic needle used for adding the precursor solution to the reactor. Switching to manual addition of the precursor solution using a 1-mL syringe resulted in successful syntheses. The Danish Medicines Agency initially approved [^18^F]FE-PE2I for human use in 2017 and, following some initial teething problems, reasonable RCYs of 7.6 ± 3.6% (*n* = 73, starting from ca. 45 GBq as the standard) were achieved. Applying the improved preparative HPLC conditions with the ascorbate buffer improved the RCYs slightly (10.8 ± 3.7%, n = 161).

**Figure 3:**
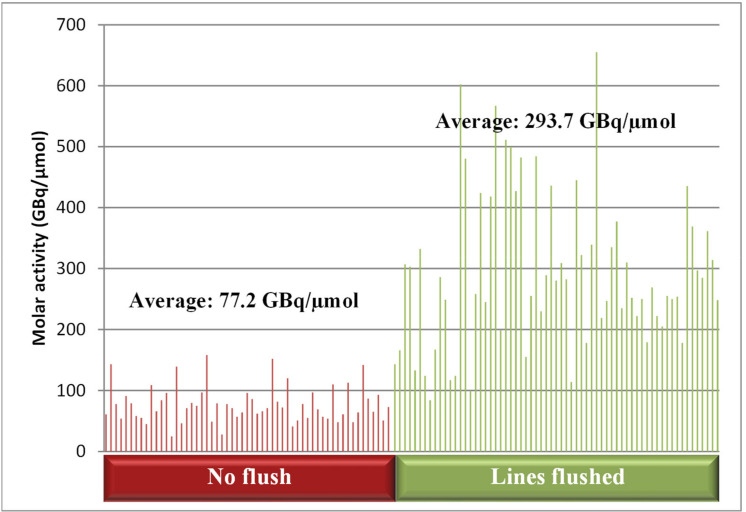
Comparison of the molar activity with and without flushing of the lines prior to synthesis.

Furthermore, it was shown that flushing the ^18^F transfer lines (Teflon tubing) from the cyclotron to the synthesis module with water prior to delivering the activity considerably increased the molar activity (A_m_) of the product (Figure 3), in line with what had previously been reported [15]. This is important since the mass of FE-PE2I and related impurities allowed per patient dose is limited to 10 µg.

#### 2.2.4. Stability Studies of the Formulated [^18^F]FE-PE2I

**Figure 4:**
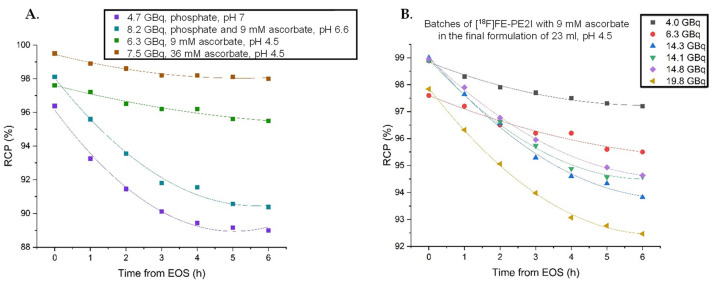
(**A**) Stability studies of the formulated [^18^F]FE-PE2I in different buffers, at different concentrations and pH. (**B**) Stability of [^18^F]FE-PE2I in 23 mL of the optimized formulation buffer over 6 h with different amounts of activity.

Stability studies over the course of 6 h were initially performed on the product which was diluted in a phosphate buffer solution, leading to the final pH of around 7. As can be seen in Figure 4A, increasing ascorbate concentrations lead to a more stable product. However, reducing the pH to around 4.5 by diluting the collected product fraction with water was shown to further increase stability for a range of activities (Figure 4B) [16]. Addition of 1-thioglycerol (0.2%) to the final product vial was found to have an additional stabilizing effect (RCP of 98% after 6 h with 16 GBq of formulated [^18^F]FE-PE2I, data not shown). However, since no toxicology data on 1-thioglycerol are available, this could not be used in humans. As far as ascorbic acid can be given intravenously in high concentrations, this is considered to be the best stabilizer for [^18^F]FE-PE2I.

### 2.3. Production of [^18^F]FE-PE2I on Synthera^®^+

**Figure 5:**
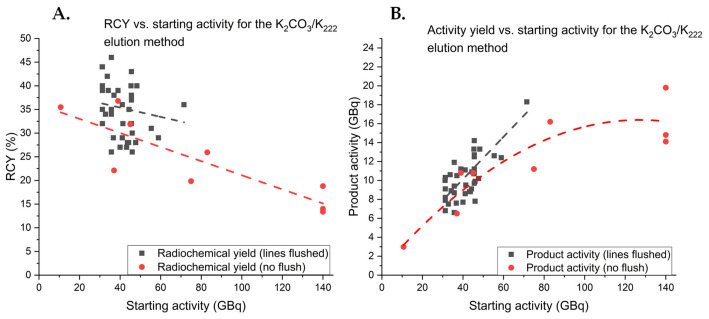
(**A**) Isolated RCYs of [^18^F]FE-PE2I in relation to the starting activity of fluorine-18. (**B**) Comparison of the activity of isolated [^18^F]FE-PE2I to the starting activity of fluorine-18.

In order to avoid manual addition of the precursor solution to the reactor to reduce staff radiation exposure, the production of [^18^F]FE-PE2I was transferred to a cassette-based system using a Synthera^®^+ HPLC (IBA). The process was fully validated with starting activities up to 140 GBq giving an average RCY of 16.9 ± 2.7% (*n* = 3). The Synthera^®^+ HPLC process is more repeatable and gives higher overall RCYs than the Scansys method. This is presumably due to minimizing losses of radioactivity in the tubing. In order to determine the optimal amount of initial activity, several syntheses were conducted using different starting activities (Figure 5).

The results show that RCYs drop significantly with higher starting activities, presumably due to increased radiolysis in the reaction solution where radioactivity is highly concentrated. Comparing the activity of isolated [^18^F]FE-PE2I to the starting activity indicated that the activity yield leveled out around 17 GBq and that increasing the starting activity further would not result in a higher activity yield. Furthermore, flushing the ^18^F transfer lines to increase A_m_ also appeared to improve the RCY of [^18^F]FE-PE2I, which was relatively stable around 35% for the 45-GBq starting activity. This phenomenon was not observed for the syntheses using a Scansys module where the yield remained unaffected. Presumably, this increase was the result of chemical impurities released from the ^18^F transfer lines in a similar manner as for the release of “cold” fluorine that affected A_m_.

Process validation: The [^18^F]FE-PE2I process on Synthera^®^+ was fully validated and summary results are shown in Table 2.

Whilst the RCYs are acceptable, an increasing clinical demand for [^18^F]FE-PE2I prompted us to try to improve the yield of the radiolabeling reaction, also in light of the increased radiolysis with increased starting activities. Recently, we published a study focusing on optimizing the preconditioning and eluting anions of the QMA cartridge to allow improved aliphatic labeling of base-sensitive tracers [11].

### 2.4. Implementing New Reaction Conditions for [^18^F]FE-PE2I

Results from the previous study showed that a variety of combinations of preconditioning and eluting anions could be used to efficiently improve the RCY of a variety of tracers and synthons. In this study, [^18^F]FE-PE2I was labeled using a PO_4_^3−^-preconditioned QMA cartridge eluted with Bu_4_NOMs, resulting in the RCC of 72.5 ± 13.1% and the RCY of 47.8 ± 7.9% (*n* = 3) for a 5-min reaction in DMSO at 120 °C [11]. Due to the potential toxicity of mesylates (OMs^−^) and no control method, a similarly high-yielding combination of anions was CO_3_^2−^-preconditioning with the Bu_4_NH_2_PO_4_ elution in either MeCN or DMSO, these conditions were chosen to continue with.

#### 2.4.1. Elution Conditions for Improved Synthesis of [^18^F]FE-PE2I

**Figure 6:**
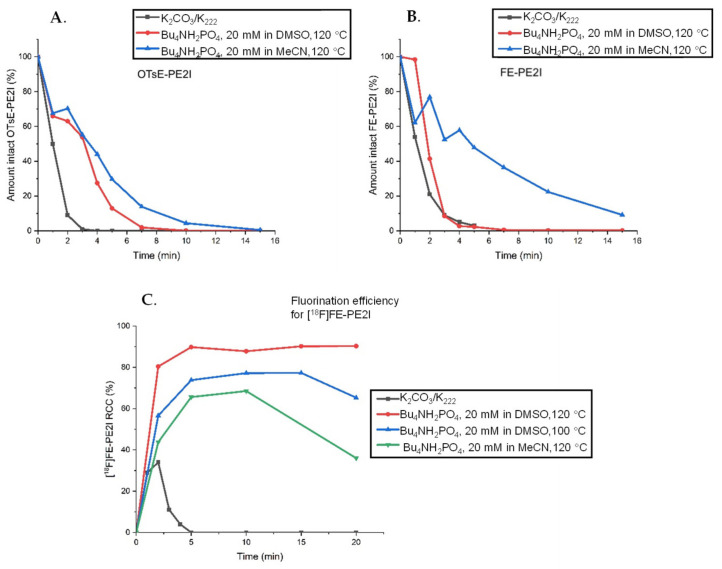
(**A**) Stability of the tosylate precursor OTsE-PE2I in the DMSO or MeCN solution at 120 °C added to the dried QMA eluate eluted by Bu_4_NH_2_PO_4_ (K_2_CO_3_/K_222_ in DMSO added for comparison). (**B**) Stability of the reference FE-PE2I in the DMSO or MeCN solution at 120 °C added to the dried QMA eluate eluted by Bu_4_NH_2_PO_4_ (K_2_CO_3_/K_222_ in DMSO added for comparison). (**C**) Radiochemical conversion for [^18^F]FE-PE2I using CO_3_^2−^-preconditioned QMA eluted by 20 mM solution of Bu_4_NH_2_PO_4_ in DMSO or MeCN.

The stability of the precursor and FE-PE2I was studied under various reaction conditions along with the ^18^F-fluorination efficiency over time (Figure 6).

The results demonstrate that the Bu_4_H_2_PO_4_ elution method provides higher stability for the precursor in both MeCN and DMSO. The stability of the reference compound in DMSO is comparable, whereas in MeCN, the reference compound is significantly more stable, with intact compound still present after 20 min of the reaction. The RCC for the ^18^F-fluorination was improved from 34% to 90% RCC using Bu_4_NH_2_PO_4_ elution compared to K_2_CO_3_/K_222_ and was relatively stable for reaction times of 5–20 min. The reason that the radiolabeled [^18^F]FE-PE2I (6C) exhibited higher stability compared to the “cold” reference reaction (6B) could be that the precursor is also present in the radiolabeling reaction. The degradation of the precursor could quench the basic environment, rendering the formed product more stable in the ^18^F reaction compared to the stability screening.

#### 2.4.2. Production of [^18^F]FE-PE2I with Improved Elution Conditions on Synthera^®^+

**Figure 7:**
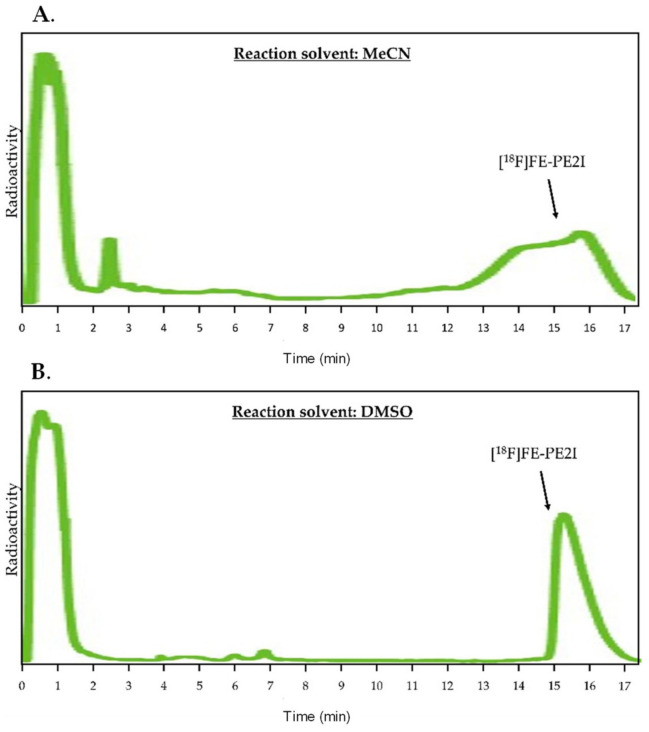
(**A**) Preparative HPLC chromatogram from the improved elution method with MeCN as the reaction solvent. (**B**) Preparative HPLC chromatogram from the improved elution method with DMSO as the reaction solvent. Both chromatograms were extracted from the synthesis reports with the time axis added for clarity. The full synthesis reports can be found in the Supplementary Materials.

**Figure 8:**
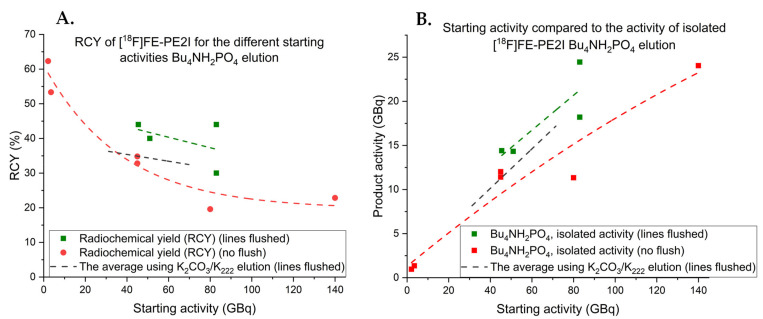
(**A**) Isolated RCY of [^18^F]FE-PE2I in relation to the starting activity of fluorine-18 with the Bu_4_NH_2_PO_4_ elution method. (**B**) Comparing the activity of isolated [^18^F]FE-PE2I to the starting activity of fluorine using the Bu_4_NH_2_PO_4_ elution method. The exponential fit from the K_2_CO_3_/K_222_ elution method is added for comparison.

The synthesis was implemented on the Synthera^®^+ module, initially testing MeCN as the reaction solvent, as this proved to be milder towards FE-PE2I during 5 min of the reaction while maintaining good RCC. However, this proved incompatible with the HPLC purification method as MeCN smeared the product over the column causing very poor isolation yields (Figure 7A). Changing the reaction solvent to DMSO provided similar radiolabeling efficiencies and allowed for efficient HPLC purification (Figure 7B). This could be due to the greater solubilizing power of DMSO. [17,18] Using DMSO as the reaction solvent generated the product with the RCY of 33 ± 2% (*n* = 3) from the 45-GBq starting activity (Figure 8).

#### 2.4.3. Limitations of the New Improved Synthesis of [^18^F]FE-PE2I on Synthera^®^+

The obtained yields from the new improved elution method were satisfactory but not as good as expected in light of the manual optimization. As autoradiolysis proved to be a limiting factor to obtain high RCYs for the previous synthesis, a series of experiments using different starting activities were conducted for the new elution method as well (Figure 8).

Lower starting activity syntheses (1–5 GBq) generated an excellent RCY (> 60%). However, autoradiolysis decreased the RCY to around 33% for the 45-GBq starting activity. Performing this synthesis with the ^18^F transfer lines flushed prior to delivery appeared to increase the RCY in the same manner as for the K_2_CO_3_/K_222_ elution method on Synthera^®^+. The highest starting activity (140 GBq) generated almost 25 GBq of [^18^F]FE-PE2I which indicates that this elution method has great potential to produce higher amounts of [^18^F]FE-PE2I. If autoradiolysis could be reduced, this method could also use its full potential enabling RCYs > 50% for high starting activity syntheses as well. To try to overcome the issues with autoradiolysis, a series of manual experiments were conducted with different concentrations of *tert*-butanol (*t*BuOH) added to the DMSO reaction solvent; it has been reported to be used as a reaction solvent for radiofluorination reactions and is known to be a good radical scavenger [19,20]. Unfortunately, after the screening of different concentrations, the reaction only tolerated a 2% (*v/v*) addition of *t*BuOH with acceptable RCC (> 50%). However, applying these conditions to full-scale 45-GBq synthesis resulted in the same final RCY as with no addition of *t*BuOH. The reason for this is currently unknown. Another option to reduce radiolysis could be to decrease the reaction time. However, decreasing the reaction time did not appear to affect the RCY regardless of the starting radioactivity (Table 3). Further experiments to resolve this issue are currently ongoing, for example, through the addition of low concentrations of 1-thioglycerol or iodine to the reaction [21].

## 3. Materials and Methods

### 3.1. General Considerations

#### 3.1.1. Reagents and Consumables

The precursor compound tosylethyl-PE2I (OTsE-PE2I) and the reference compound FE-PE2I were supplied by PharmaSynth AS (Tartu, Estonia). Acetonitrile (MeCN, anhydrous, 99.8%), 1,10-diaza-4,7,13,16,21,24-hexaoxabicyclo[8,8,8]-hexacosane (Kryptofix 222, K_222_), potassium carbonate, dimethyl sulfoxide (DMSO; anhydrous, 99.8%), tetraethylammonium hydrocarbonate (Et_4_NHCO_3_; ≥95.0%) and monobasic tetrabutylammonium phosphate (Bu_4_NH_2_PO_4_; puriss., ≥99.0%) were purchased from Sigma-Aldrich (St. Louis, MO, USA). Water for injections (Ph. Eur.) was supplied by Thermo Fisher Scientific (Roskilde, Denmark), ascorbic acid by Fagron A/S (Copenhagen, Denmark), ethanol (96%; Ph. Eur.) by Region Hovedstadens Apotek (Herlev, Denmark), nitrogen gas by Strandmøllen (Klampenborg, Denmark). Preconditioned QMA cartridges (CO_3_^2−^ as the counter ions) were supplied by ABX advanced biochemical compounds GmbH (Radeburg, Germany). Preconditioned Chromafix PS-30 cartridges (HCO_3_^−^ as the counter ions) were supplied by IBA RadioPharma Solutions (Louvain-la-Neurve, Belgium). All the reagents, solvents, cartridges and filters were used as received from the commercial suppliers.

#### 3.1.2. [^18^F]Fluoride

The ^18^O(p,n)^18^F reaction with proton irradiation of ^18^O-enriched water (98%, Rotem Industries Ltd., Medical Imaging, Dimona, Israel) using a Scanditronix MC-32 (16 MeV) or Eclipse TM HP (11 MeV) cyclotron was used to produce [^18^F]fluoride at Copenhagen University Hospital, Rigshospitalet. The [^18^F]fluoride in ca. 2.5 mL ^18^O-enriched water was collected in a small V-shaped vial and then automatically transferred to a QMA cartridge (preconditioned with a specified counter ion and rinsed with water for injections).

### 3.2. General Description of the Manual Optimization of Reaction Conditions

#### 3.2.1. General Method for Stability Studies of FE-PE2I and OTsE-PE2I

Stability of the reference compounds FE-PE2I and OTsE-PE2I was monitored under various conditions. The specified anion exchange cartridge was eluted into a V-shaped 4-mL vial with a corresponding eluting anion and evaporated to dryness under the N_2_ flow at 100 °C to mimic the real production process. Solutions of FE-PE2I (50 µg) or OTsE-PE2I (1.0 mg) in the reaction solvent (1 or 0.6 mL) were added and the mixtures were heated to the specified reaction temperature. The lower concentration of FE-PE2I was used to mimic the low amounts of [^18^F]FE-PE2I formed in the radiolabeling reaction while still allowing for adequate detection and quantification. Small samples were removed at specified time intervals using a 1-mL syringe. Ten microliters of the solution were moved to an HPLC vial containing 40 µL 25 mM ascorbic acid (pH 4.3) and analyzed using HPLC to quantify the remaining compound by integrating the peaks corresponding to the reference or the precursor at 220 nm.

#### 3.2.2. General Method for Manual Radiolabeling Reactions of [^18^F]FE-PE2I

An aliquot of aqueous [^18^F]fluoride was trapped on a preconditioned anion exchange cartridge. The activity was eluted by the specified eluting anion solution into a V-shaped 4-mL vial that was subsequently evaporated to dryness under the N_2_ flow at 100 °C. To the dried [^18^F]fluoride, the precursor OTsE-PE2I (1 mg) was added in the specified reaction solvent (1 or 0.6 mL), and the samples were taken at specified time intervals using a 1-mL syringe and diluted in 25 mM ascorbic acid buffer (pH 4.3). The samples were analyzed by analytical HPLC and TLC.

### 3.3. Detailed Description of the Manual Optimization of Reaction Conditions; Details for Stability Studies of FE-PE2I and OTsE-PE2I and the Manual Fluorination Reaction of [^18^F]FE-PE2I

The experimental details for the stability studies with different elution methods for FE-PE2I and OTsE-PE2I are shown in Table 4 and Table 5.

### 3.4. Process Description of the Production of [^18^F]FE-PE2I on a Scansys Synthesis Module

**Figure 9:**
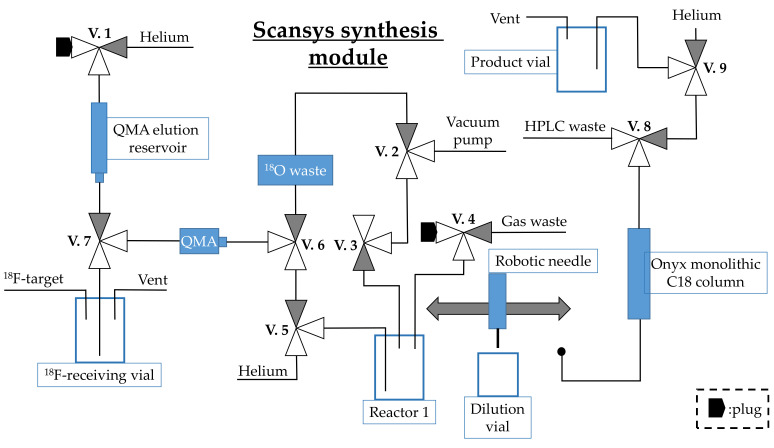
Setup of the Scansys synthesis module used for synthesis of [^18^F]FE-PE2I.

The setup of the Scansys synthesis module is depicted in Figure 9. Aqueous [^18^F]F^−^ target water was delivered to the ho tcell and collected in the ^18^F-receiving vial. When all the activity had been collected, the aqueous [^18^F]F^−^ solution was trapped on a CO_3_^2−^-preconditioned QMA cartridge by opening V.6 and V.2, and [^18^O]H_2_O was collected in the ^18^O waste. QMA was eluted by 1 mL K_2_CO_3_/K_222_ (7.8/15.6 µmol) dissolved in H_2_O/MeOH (18:82) by opening valves V.1, V.7, V.5 and V.4 into the V-shaped 4-mL vial in reactor 1. The eluate was concentrated to dryness under He steam (4.1 mL/min) and vacuum at 100 °C by opening V.7, V.5, V.4 and V.3 for 1120 s. A 1-mL syringe containing 1 mg OTsE-PE2I (1.6 µmol) dissolved in 0.6 mL dry DMSO was used to add the precursor to the dried ^18^F-floride in reactor 1 by hand with V.4 open. The reaction was incubated at 135 °C for 2 min, after which reactor 1 was cooled to 30 °C. The reaction in reactor 1 was diluted with 3.7 mL ascorbate/ascorbic acid buffer (25 mM, pH 4.3) from the dilution vial using a robotic needle and opening V.4. The solution was mixed by flushing it up and down in the robotic needle and was then transferred and injected to the preparative HPLC. HPLC purification was performed using a 10 × 150 mm monolithic column (Onyx C18, Phenomenex) eluted with 18% ethanol in the sodium ascorbate/ascorbic acid buffer (25 mM, pH 4.3), flow rate: 5.5 mL/min. The fraction corresponding to [^18^F]FE-PE2I was collected over 90 s (RT: 17 min). The flow was diverted through a 0.22-µm sterile filter into a 20-mL borosilicate glass vial (product vial) containing 15 mL water for injections by opening V.8. After the collection was complete, the remaining product in the tubing was eluted by helium by opening V.9 which was left on for 30 s to obtain a well-mixed product by bubbling helium through the solution.

### 3.5. Process Description of the Production of [^18^F]FE-PE2I on a Synthera^®^+ Synthesis Module

**Figure 10:**
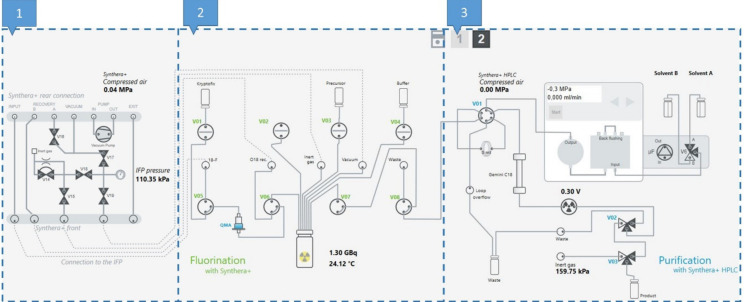
Setup of the Synthera^®^+ synthesis module used for the synthesis of [^18^F]FE-PE2I. Part 1: gas flow and vacuum regulation. Part 2: liquid transfer, heating and activity measurements. Part 3: preparative HPLC purification and collection of the product.

The setup of the Synthera^®^+ synthesis module is depicted in Figure 10. The same setup was used for the two elution conditions.

Aqueous [^18^F]F^−^ target water was delivered to the hot cell and collected in the ^18^F-receiving V-shaped 5-mL vial. When all the activity had been collected, the aqueous [^18^F]fluoride solution was trapped on a CO_3_^2−^-preconditioned QMA cartridge by applying vacuum over V05 and V06 for 45 s. The QMA was then eluted using 1 mL K_2_CO_3_/K_222_ (7.8/15.6 µmol) dissolved in H_2_O/MeOH (18:82) (A) or 0.6 mL Bu_4_NH_2_PO_4_ (20 µmol) in MeCN/H_2_O (50:50) from the Kryptofix vial (B) by applying vacuum to the 10-mL reactor. The ^18^F eluate was then concentrated to dryness under the N_2_ flow at 300 kPa at 110 °C for 7 min. The temperature and pressure in the reactor were then lowered to 90 °C and 3 kPa, respectively, for 45 s by closing V15, followed by 10 purging steps where the reactor was flushed with N_2_ for 2 s followed by 2 s of vacuum. This cycle was repeated three times, resulting in complete removal of the eluting solvent. The precursor TsOE-PE2I (1 mg in 0.6 mL DMSO (A) or 1.0 mL DMSO (B)) was added from the precursor vial by applying vacuum over the reactor. The reaction was then incubated at 135 °C for 2 min (A) or 120 °C for 5 min (B), after which the heating was removed from the reactor for 1 min. The reaction mixture was diluted with 3.7 mL ascorbate/ascorbic acid buffer (25 mM, pH 4.3) from the buffer vial by applying vacuum over the reactor. The diluted reaction mixture was then transferred to the HPLC loop over V07 and V08 by applying the N_2_ flow on the reactor with a 0.22-µm sterile filter that cut off the flow once all the liquid had been transferred. HPLC purification was performed using a 10 × 150 mm monolithic column (Onyx C18, Phenomenex) eluted with the sodium ascorbate/ascorbic acid buffer (25 mM, pH 4.3) containing 18% ethanol, flow rate: 6 mL/min. The fraction corresponding to [^18^F]FE-PE2I was collected over 2 min (RT: 17 min). The flow was diverted to a 30-mL glass vial (product vial) containing 15 mL water for injections by opening valve V02. After the collection was complete, the remaining product in the tubing was eluted by the N_2_ flow by opening V03 which was left on for 2 min to obtain a well-mixed product by bubbling nitrogen through the solution.

### 3.6. Quality Control

Product specifications for [^18^F]FE-PE2I and analytical methods are summarised in Table 6.

#### 3.6.1. HPLC

HPLC analysis was performed using an Ultimate 3000 system (Thermo Fisher Scientific) with a diode-array UV detector and a Geiger–Müller tube-based radiodetector (Scansys Laboratorieteknik, Denmark). A C18 reversed-phase column (Accucore, 2.6 µm, C18, 100 Å, 150 × 4.6 mm, Thermo Fisher Scientific) was eluted with 75% acetonitrile in 25 mM sodium phosphate buffer (pH 6.2); injection volume, 50 µL; flow rate, 1.5 mL/min; on-line UV (220 nm) and radioactivity detection.

#### 3.6.2. GC Analysis

GC analysis was performed using a GC-2014 system (Shimadzu); column: Zebron ZB-WAX, 30 m × 0.53 mm, 1 µm (Phenomenex); temperature gradient: 70 °C, 0–4 min; 70–230 °C, 4–10.6 min; flow rate: 4.7 mL/min (helium); injection volume: 0.25 µL; split ratio: 5; detection: FID; RT: methanol, 2.4 min; ethanol, 2.6 min; acetonitrile, 3.5 min; DMSO, 9.2 min.

#### 3.6.3. TLC Analysis

For the radiolabeling optimization experiment, TLC was performed using silica gel 60 F254 (Merck) TLC plates with an eluent consisting of (33:67) heptane/ethyl acetate (*v/v*) (rf: 0.6). The radioactive traces on the plates were detected using photostimulated luminescence plates (PSP) (Perkin Elmer) by incubating the TLC plates on the PSP for 5 min. The PSP were read in a cyclone reader (Cyclone Plus Phosphor Imager, PerkinElmer, Inc., Waltham, Massachusetts, USA) and analyzed using Optiquant.

#### 3.6.4. Other Analyses

The product’s pH was determined using a calibrated pH-meter (Knick Portamess^®^ 913 pH with a Hamilton^®^ MiniTrode pH electrode). Analysis for residual Kryptofix 222 was performed using standard color spot test procedures [22]. Radionuclidic purity measurements were performed using a high-purity germanium detector (Canbarra) 48 h after the end of synthesis to allow decay of all ^18^F. Two-milliliter samples were measured over 12 h. Bacterial endotoxin levels were determined using a Nexgen PTS reader (Charles River).

## 4. Conclusions

At first glance, the synthesis of [^18^F]FE-PE2I appears to be fairly straightforward as it is one-step aliphatic radiofluorination [10]. However, scaling the synthesis from the reported 5-GBq starting activity more than 20-fold, radiolysis both during the reaction and for the formulated product was observed. We also observed strong sensitivity of the precursor and the product towards the basic conditions which lead to decomposition. The optimization of the elution conditions using Bu_4_NH_2_PO_4_ resulted in a major improvement of the RCC from ~35% to > 80%, which then translated to an isolated RCY of up to 62% for low-activity synthesis (< 40 GBq). For higher-activity syntheses (> 40 GBq), autoradiolysis occurred and reduced the RCY. However, up to 25 GBq of [^18^F]FE-PE2I could be produced from 80 GBq of the starting activity with the RCY of 40% using Bu_4_NH_2_PO_4_ elution with the lines flushed. Currently, the K_2_CO_3_/K_222_ elution method is validated for GMP-compliant clinical production and provides robust synthesis with the average RCY of 35% (10.5 GBq) from the 45-GBq starting activity using a cassette-based Synthera^®^+ synthesis module. The Bu_4_NH_2_PO_4_ elution method holds even greater potential, averaging 42% RCY for the 45-GBq starting activity and could, with a suitable method for reducing radiolysis, further increase the activity yield of [^18^F]FE-PE2I, satisfying the increased clinical demand for this tracer.

## Figures and Tables

**Table 1:** Summary of the validation data for the HPLC analysis of [^18^F]FE-PE2I.

Test Parameter	Acceptance Criteria		Result	
Specificity	Resolution of >2 between the peaks		Resolution (impurity at the RT of 3.4 and FE-PE2I) = 2.6	
Linearity	0.25–1.25 µg/mL (five concentrations in triplicate)	R^2^ > 0.995	R^2^ > 0.998	
Repeatability	10 repetitions (1 µg/mL standard)	RSD ≤ 5%	RSD = 0.48%	
Accuracy	Recovery 90–110% (matrix spiked with FE-PE2I; five different concentrations)		Concentration	Recovery
			0.1 µg/mL 0.2 µg/mL 0.3 µg/mL 0.4 µg/mL 0.5 µg/mL	97% 96% 106% 103% 101%
Limit of detection	S/N * ratio ≥ 3		0.01 µg/mL	
Limit of quantification	S/N ratio ≥ 10		0.05 µg/mL	

* Signal-to-noise; RT = retention time.

**Table 2:** Details for process validation of [^18^F]FE-PE2I produced on a Synthera^®^+ synthesis module.

Test	Specification	Batch Results		
Batch No.		PE2I-200513-2	PE2I-200514-2	PE2I-200520-2
Radioactivity	0.2–20 GBq at EOS	14.1 GBq	14.8 GBq	19.8 GBq
Radiochemical yield (DC)	Reported value	13%	14%	20%
[^18^F]Fluoride	≤5%	0.1%	0.2%	0.2%
Other ^18^F impurities	≤5%	1.0%	0.9%	2.0%
Radiochemical purity at EOS	≥96%	98.9%	99.0%	97.8%
Radiochemical purity after 6 h	≥90%	94.6%	94.6%	92.5%
FE-PE2I	≤1.00 µg/mL	0.45 µg/mL	0.44 µg/mL	0.28 µg/mL
Specified impurity (RT of 3.4 min)	≤1.00 µg/mL	0.15 µg/mL	0.20 µg/mL	0.13 µg/mL
Total unspecified impurities	1.00 µg/mL	n.d.	n.d.	n.d.
Total FE-PE2I and impurities	≤1.00 µg/mL	0.60 µg/mL	0.64 µg/mL	0.41 µg/mL
Molar radioactivity	Reported value	619 GBq/µmol	664 GBq/µmol	1393 GBq/µmol

n.d.—none detected.

**Table 3:** Data from different efforts to try to overcome the problem of radiolysis with high-activity syntheses of [^18^F]FE-PE2I. Top: manual experiments with low activity to see how much *t*BuOH is tolerated in the reaction solvent. Bottom: isolated RCY with either *t*BuOH added or the reaction time reduced (lines not flushed).

Solvent	RCC, 2 min	RCC, 5 min	RCC, 10 min	RCC, 20 min
DMSO, 10% *t*BuOH	8.5%	26.8%	30.4%	30.8%
DMSO, 5% *t*BuOH	13.6%	36.5%	42.1%	46.8%
DMSO, 2% *t*BuOH	37.9%	75.6%	77.4%	83.8%
**Results of full synthesis**	**DMSO, 2% *t*BuOH, 5 min reaction time**	**DMSO, 2 min reaction time**	**DMSO, 3.5 min reaction time**	**DMSO, 5 min reaction time**
Isolated RCY (45-GBq starting activity)	32.8%	32.0%	-	34.8%
Isolated RCY (140-GBq starting activity)	-	23.0%	20.8%	22.9%

**Table 4:** Specified details for the first manual stability tests of FE-PE2I and OTsE-PE2I and the manual fluorination reaction of [^18^F]FE-PE2I. * Two equivalents of K_222_ used compared to K_2_CO_3_.

Sample	K_2_CO_3_/K_222_	Et_4_NHCO_3_ 75 mM	Et_4_NHCO_3_ 50 mM	Et_4_NHCO_3_ 25 mM	Et_4_NHCO_3_ 25 mM
Reaction temperature	140 °C	140 °C	140 °C	140 °C	120 °C
Reaction solvent	DMSO (0.6 mL)	DMSO (0.6 mL)	DMSO (0.6 mL)	DMSO (0.6 mL)	DMSO (0.6 mL)
Anion exchange cartridge	QMA	PS-30	PS-30	PS-30	PS-30
Preconditioning anion	CO_3_^2−^	HCO_3_^−^	HCO_3_^−^	HCO_3_^−^	HCO_3_^−^
Eluting anion	K_2_CO_3_/K_222_ *	Et_4_NHCO_3_	Et_4_NHCO_3_	Et_4_NHCO_3_	Et_4_NHCO_3_
Eluting anion concentration	1.1 mg (7.8 µmol)	14.3 mg (75 µmol)	9.6 mg (50 µmol)	4.8 mg (25 µmol)	4.8 mg (25 µmol)
Eluting anion solvent	H_2_O/MeOH (180 µL/820 µL)	H_2_O/MeOH (180 µL/820 µL)	H_2_O/MeOH (180 µL/820 µL)	H_2_O/MeOH (180 µL/820 µL)	H_2_O/MeOH (180 µL/820 µL)

**Table 5:** Specified details for the second manual stability tests of FE-PE2I and OTsE-PE2I and the manual fluorination reaction of [^18^F]FE-PE2I.

Sample	Bu_4_NH_2_PO_4_	Bu_4_NH_2_PO_4_	Bu_4_NH_2_PO_4_
Reaction temperature	120 °C	100 °C	120 °C
Reaction solvent	DMSO (1.0 mL)	DMSO (1.0 mL)	MeCN (1.0 mL)
Anion exchange cartridge	QMA	QMA	QMA
Preconditioning anion	CO_3_^2−^	CO_3_^2−^	CO_3_^2−^
Eluting anion	Bu_4_NH_2_PO_4_	Bu_4_NH_2_PO_4_	Bu_4_NH_2_PO_4_
Eluting anion concentration	6.8 mg (20.0 µmol)	6.8 mg (20.0 µmol)	6.8 mg (20.0 µmol)
Eluting anion solvent	H_2_O/MeCN (500 µL/500µL)	H_2_O/MeCN (500 µL/500 µL)	H_2_O/MeCN (500 µL/500 µL)

**Table 6:** Specifications and analytical methods for the quality control of [^18^F]FE-PE2I.

Test	Specifications	Analytical Methods
Identification of [^18^F]FE-PE2I	Radioactive half-life: 105–115 min Gamma spectrum shows only the 511 and 1022 keV peaks. The labeled product corresponds in RT to an authentic reference standard of FE-PE2I	T_1/2_ measurement using a dose calibrator Gamma spectrum using a NaI well counter Product ID using HPLC
Radioactivity	0.2–20 GBq at EOS	Dose calibrator
Volume	32 ± 1 mL	Visual check
Appearance	Clear and colorless solution free from visible particulates or cloudiness	Visual inspection
pH	4.0–5.0	pH-meter
Residual Kryptofix	< 0.075 mg/mL	Color spot test
Free [^18^F]fluoride	≤ 5%	HPLC with a radiodetector
Other ^18^F-labeled impurities	≤ 5%	HPLC with a radiodetector
Radiochemical purity (at EOS)	≥ 96%	HPLC with a radiodetector
Radiochemical purity after 6 h (end of the shelf life)	≥ 90%	HPLC with a radiodetector
FE-PE2I content	≤ 1.00 µg/mL	HPLC (UV, 220 nm)
Specified impurity (HPLC, RT of 3.4 min)	≤ 1.00 µg/mL	HPLC (UV, 220 nm)
Total unspecified organic impurities	≤ 1.00 µg/mL	HPLC (UV, 220 nm)
Total FE-PE2I and organic impurities	≤ 1.00 µg/mL(maximum injected dose of 10 µg)	HPLC (UV, 220 nm)
Radionuclidic purity	≥ 99.9% (< 0.1% radionuclidic impurities)	Gamma spectrum using an HPGe detector after the complete decay of fluorine-18 (minimum 48 h after EOS)
Ethanol	3–7% (*w/v*)	Gas chromatography
Acetonitrile	≤ 273 ppm	Gas chromatography
Methanol	≤ 2000 ppm	Gas chromatography
DMSO	≤ 3333 ppm	Gas chromatography
Microbiology	Passes the test for sterility (Ph. Eur.)	Test for sterility (Ph. Eur.), filtration method
Bacterial endotoxins	≤ 0.5 EU/mL	Quantitative LAL analysis (Ph. Eur.)
Microbiology	Bioburden: < 10 CFU/100 mL	Membrane filtration and media growth

## Data Availability

The data presented in this study are available in article and Supplementary Materials.

## Supplementary Materials

The following are available online at https://www.mdpi.com/article/10.3390/ph14070601/s1.
