# Supplementary material for: Fully Automated GMP-Compliant Synthesis of [18F]FE-PE2I
Source: Pharmaceuticals (Basel). 2021 Jun 22;14(7):601. doi: 10.3390/ph14070601 (PMC8308591; doi:10.3390/ph14070601)
Supplement: Supplementary file 1 [file pharmaceuticals-14-00601-s001.zip › pharmaceuticals-1255087-SI.pdf]

pharmaceuticals

Supporting information

Supporting information: Fully automated GMP compliant synthesis of ^{18}F-FE-PE2I

Klas Bratteby[1,2,3,†], Charlotte Lund Denholt[2,†], Szabolcs Lehel[2], Ida Nyman Petersen[2], Jacob Madsen[2], Maria Erlandsson[3], Tomas Ohlsson[3], Matthias Manfred Herth[1,2] and Nic Gillings[2,*]

1 Department of Drug Design and Pharmacology, University of Copenhagen, Jagtvej 160, 2100 Copenhagen, Denmark; klas.bratteby@sund.ku.dk (KB); matthias.herth@sund.ku.dk (MMH).
2 Department of Clinical Physiology Nuclear Medicine & PET, Rigshospitalet, Blegdamsvej 9, 2100 Copenhagen, Denmark; charlotte.lund.denholt@regionh.dk (CLD); szabolcs.lehel@regionh.dk (SL); ida.nymann.petersen@regionh.dk (INP); jacob.madsen@regionh.dk (JM); nicholas.mathew.gillings@regionh.dk (NG).
3 Department of Radiation Physics, Skåne University Hospital, Barngatan 3, 22242 Lund, Sweden; tomas.ohlsson@skane.se (TO); maria.n.erlandsson@skane.se (ME).
† These authors contributed equally to this work.
* Correspondence: ngil0001@regionh.dk; Tel.: +45 35451678 (NG)

Chromatograms and data from stabillity studies of OTsE-PE2I

Stabillity studies were conducted on the precursor (OTsE-PE2I) and reference (FE-PE2I) by adding solutions of the respective compounds to dried eluates from the QMA. The same experiments were performed for elutions using K_2CO_3/K_{222}, Et_4NHCO_3 and $Bu_4NH_2PO_4$ Some examples of the analytical HPLC chromatograms can be seen in Figure S1.

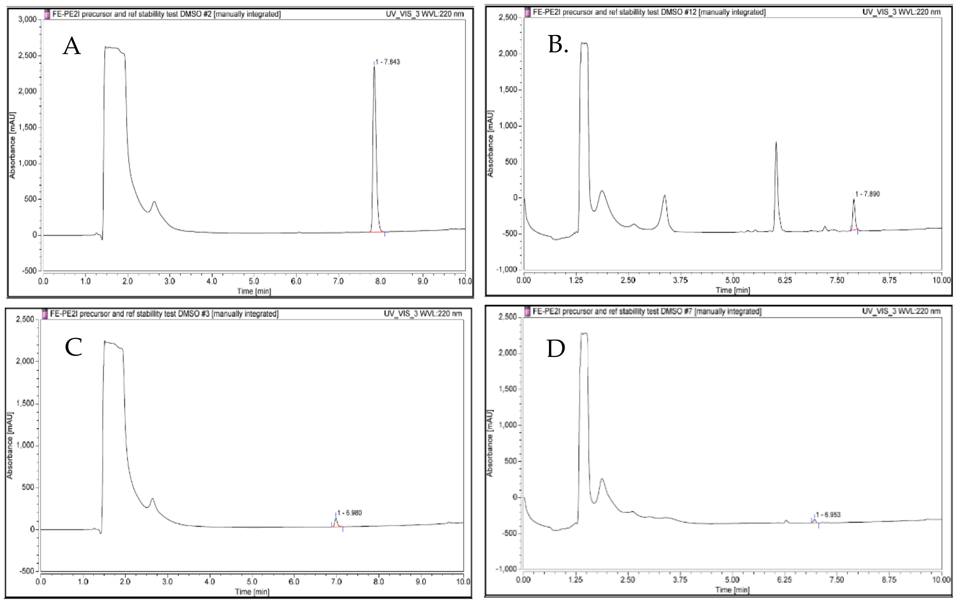

Figure S1. Examples of anlytical HPLC chromatograms to evaluate the stability of the tosylate precursor (OTsE-PE2I) and reference (FE-PE2I) dissolved in DMSO at 120 °C added to dried QMA eluates using $Bu_4NH_2PO_4$ (20 mM, 1 mL) for elution. The integrated peaks correspond to the identfied respective compound and remaining concentration at later time points (2-15 min) where calculated as a percentage of t = 0 min. The chromatograms display OTsE-PE2I dissolved in DMSO at t = 0 min (A.) and t = 10 min (B.) as well as FE-PE2I at t = 0 min (C.) and t = 2 min (D.).

Synthesis reports for synthesis using the K_2CO_3/K_{222} and $Bu_4NH_2PO_4$-elution methods starting from 45 GBq starting activity on Synthera® + synthesis module.
