# Supplementary material for: Fully Automated GMP-Compliant Synthesis of [18F]FE-PE2I
Source: Pharmaceuticals (Basel). 2021 Jun 22;14(7):601. doi: 10.3390/ph14070601 (PMC8308591; doi:10.3390/ph14070601)

Synthesis report

SW: Version 1.1 | October 2018

Document id: S2005270363 Batch Nr: PE2I-200527-2

Start at 5/27/2020 9:20:44 AM
End at 5/27/2020 9:41:17 AM
Duration: 0:20:32

User: User Default (Supervisor)
Level: Supervisor

[18F]Fluorine
FE-PE2I
Status: Synthesis completed

Start activity:
End activity:
Estimated yield: % n.d.c.

Recipe: PE2I riget (v2)
Script: PE2I-riget (v6)

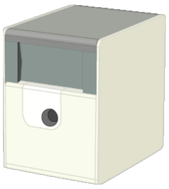

Synthera+
IBA SN: SM182802
Site Id: Synthera+ #1
HW: 1246398

IFP: 2-2560-1021062547364
Status: Single use IFP found
Consumable:
NA

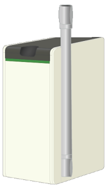

Synthera+ HPLC
IBA SN: HP183703
Site Id: HPLC #1
HW: 1250402

Comments:

Author	Manager	Quality
Name:	Name:	Name:
Date:	Date:	Date:
Comment:	Comment:	Comment:

Synthesis report

Synthesis report

SW: Version 1.1 | October 2018

Document id: S2005270363 Batch Nr: PE2I-200527-2

Synthera+ (SM182802) - Details

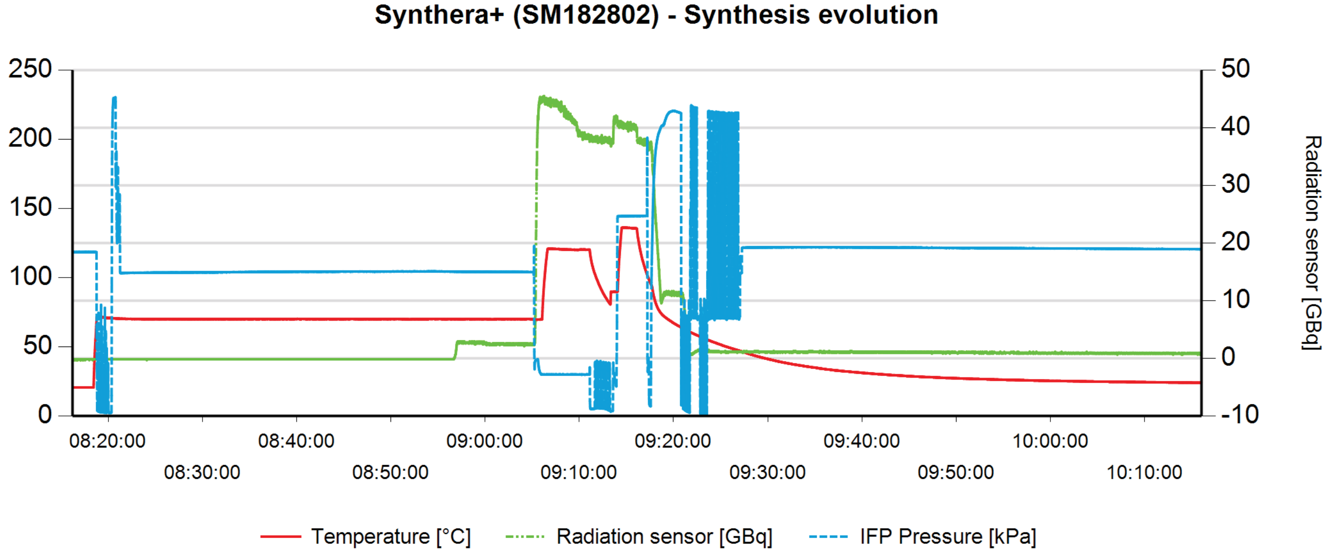

9:27:27 AM	Final sequence status: Completed		
8:16:13 AM	*Command: Load*		
8:17:51 AM	*Command: Start*		

	Sequence evolution	
8:18:04 AM	**Initialization**	**Completed**
8:18:26 AM	**Valve rotation test**	**Completed**
8:18:44 AM	**Temperature test and pre-heat**	**Completed**
8:18:43 AM	*Action: Signal report*	Temperature value: 63,59 °C
8:20:01 AM	**Purging of the IFP**	**Completed**
8:20:20 AM	**Vacuum test**	**Completed**
8:20:07 AM	*Action: Signal report*	IFP Pressure value: 2,21 kPa
8:20:19 AM	*Action: Signal report*	IFP Pressure value: 2,43 kPa
8:20:44 AM	**Pressure test**	**Completed**
8:20:31 AM	*Action: Signal report*	IFP Pressure value: 226,03 kPa
8:20:44 AM	*Action: Signal report*	IFP Pressure value: 229,32 kPa
8:39:39 AM	**Test tube 1 - 2 - 3 - 4**	**Completed**
9:00:02 AM	**WAITING FOR ACTIVITY**	**Completed**
9:00:01 AM	*Action: Resume (WD;USR;;)*	
9:05:07 AM	**Trapping Fluorine-18**	**Completed**
9:06:03 AM	**Elution Fluorine-18**	**Completed**
9:06:02 AM	*Action: Signal report*	Radiation sensor value: 44,88 GBq
9:13:34 AM	**Drying Fluorine-18**	**Completed**
9:11:21 AM	*Action: Signal report*	IFP Pressure value: 5,13 kPa
9:13:16 AM	*Action: Signal report*	IFP Pressure value: 4,12 kPa

Synthesis report

Document id: S2005270363 Batch Nr: PE2I-200527-2

9:14:02 AM	**Precursor to reactor**	**Completed**
9:17:08 AM	**Labelling**	**Completed**
9:17:39 AM	**Buffer to reactor**	**Completed**
9:20:44 AM	**Transfer to HPLC**	**Completed**
9:27:14 AM	**Purging of the pump**	**Completed**
9:27:26 AM	**Re-initialization**	**Completed**

Synthesis report

Document id: S2005270363 Batch Nr: PE2I-200527-2

Synthera+ HPLC (HP183703) - Details

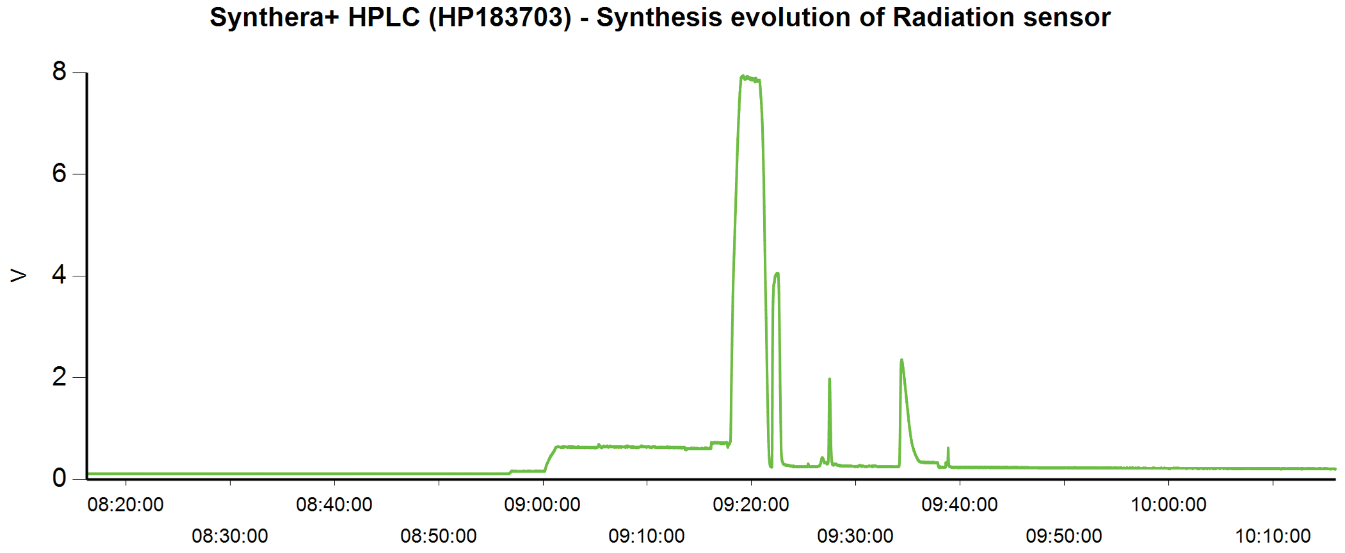

9:53:30 AM	Final sequence status: Completed		
8:16:13 AM	*Command: Load*		
8:17:51 AM	*Command: Start*		

	Sequence evolution	
8:34:04 AM	**Pre-cleaning**	**Completed**
8:39:39 AM	**Conditioning**	**Completed**
9:18:10 AM	**Ready to start**	**Completed**
9:20:44 AM	**Wait transfer from Synthera+**	**Completed**
9:23:47 AM	**Purification - Collection**	**Completed**
9:34:18 AM	**Press play to collect product**	**Completed**
9:34:12 AM	*Action: Resume (WD;V02;1;)*	
9:41:18 AM	**Press play to stop collection**	**Completed**
9:36:15 AM	*Action: Resume (WD;V02;0;)*	
9:53:28 AM	**Cleaning**	**Completed**

Synthesis report

SW: Version 1.1 | October 2018

Document id: S2005270363 Batch Nr: PE2I-200527-2

Synthera+ (SM182802) - Synthesis evolution of Temperature

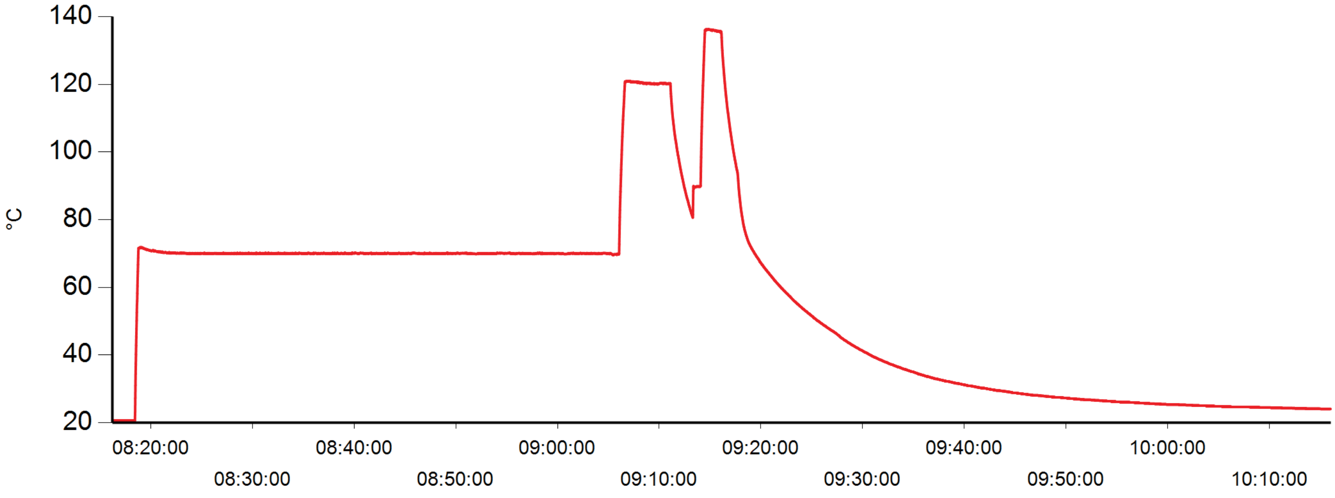

Synthera+ (SM182802) - Synthesis evolution of Radiation sensor

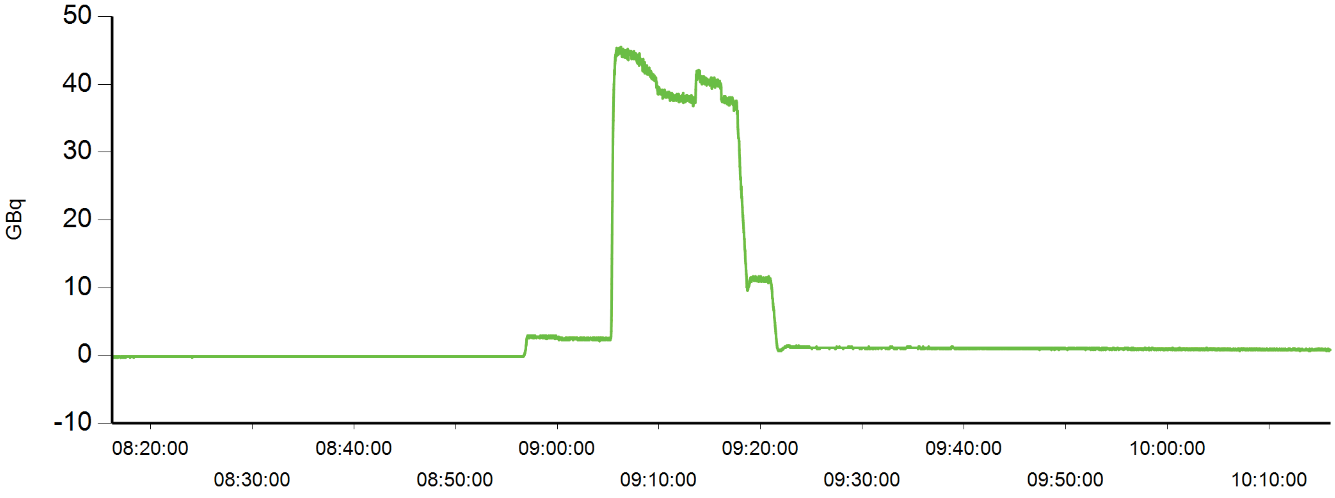

Synthera+ (SM182802) - Synthesis evolution of Compressed air

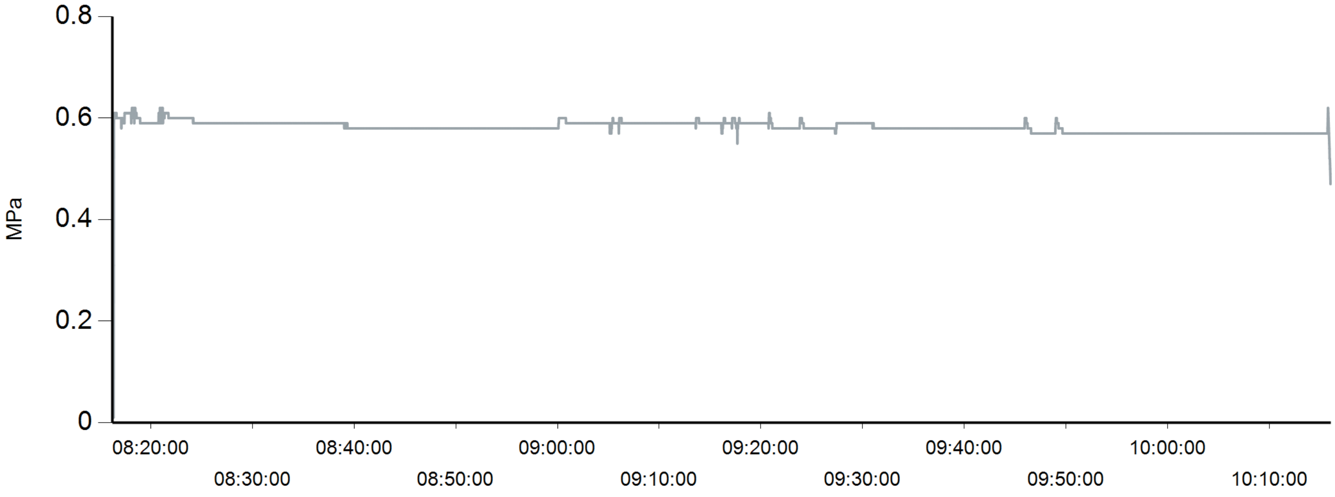

Synthesis report

Document id: S2005270363   Batch Nr: PE2I-200527-2

Synthera+ (SM182802) - Synthesis evolution of IFP Pressure

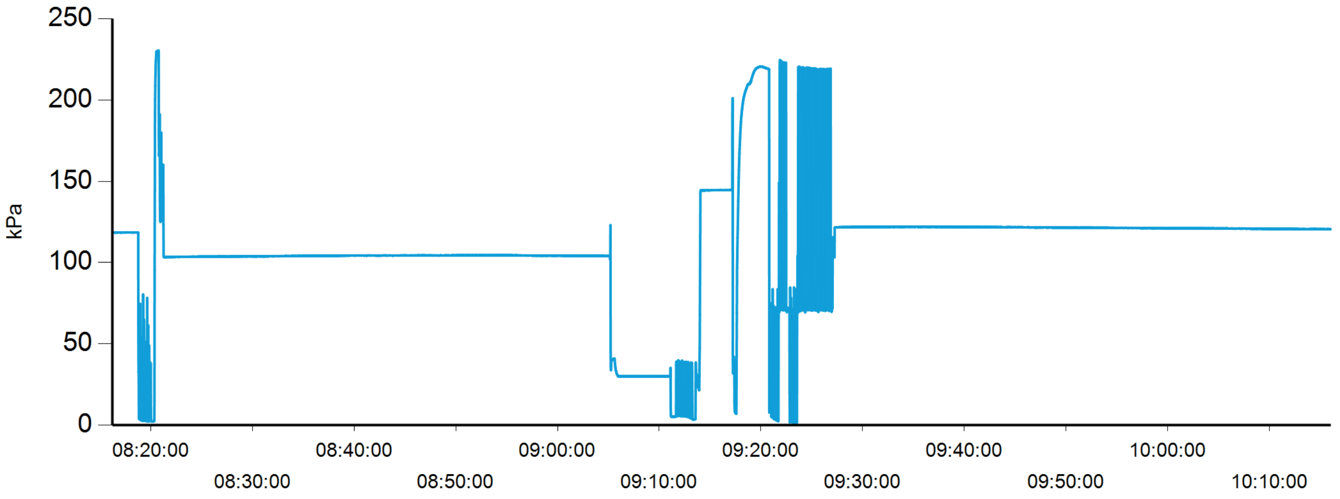

Synthera+ (SM182802) - Synthesis evolution of Aux. Signal

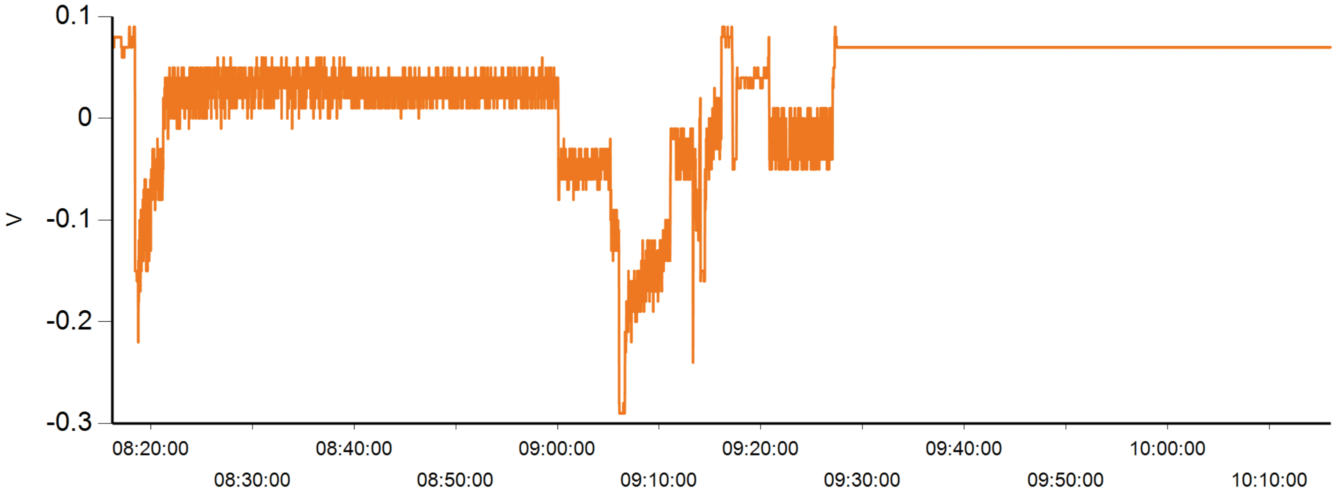

Synthesis report

SW: Version 1.1 | October 2018

Document id: S2005270363 Batch Nr: PE2I-200527-2

Synthera+ HPLC (HP183703) - Synthesis evolution of Radiation sensor

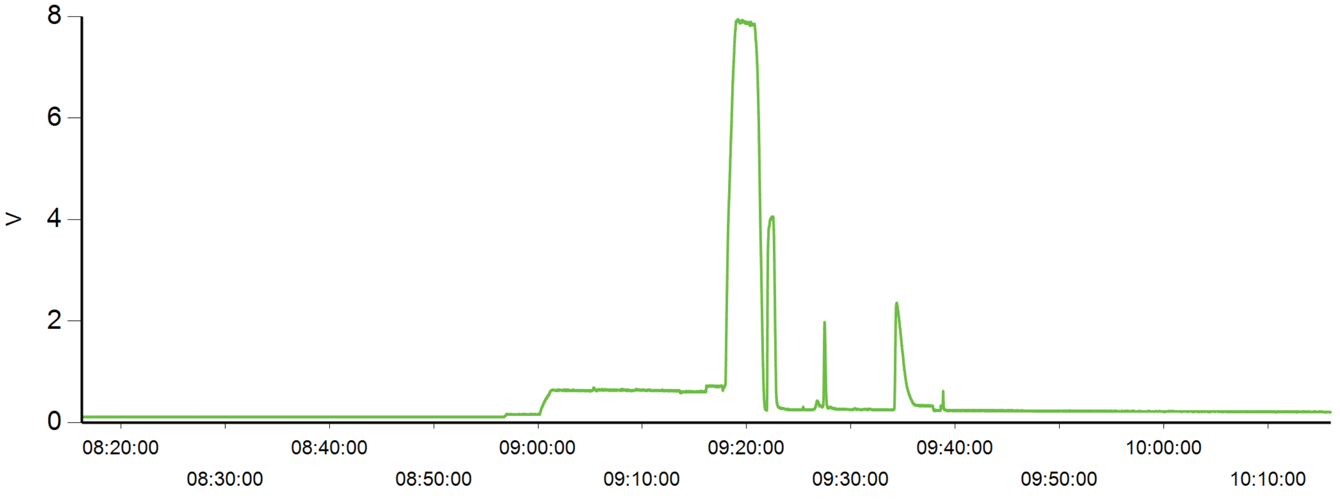

Synthera+ HPLC (HP183703) - Synthesis evolution of Inert gas pressure

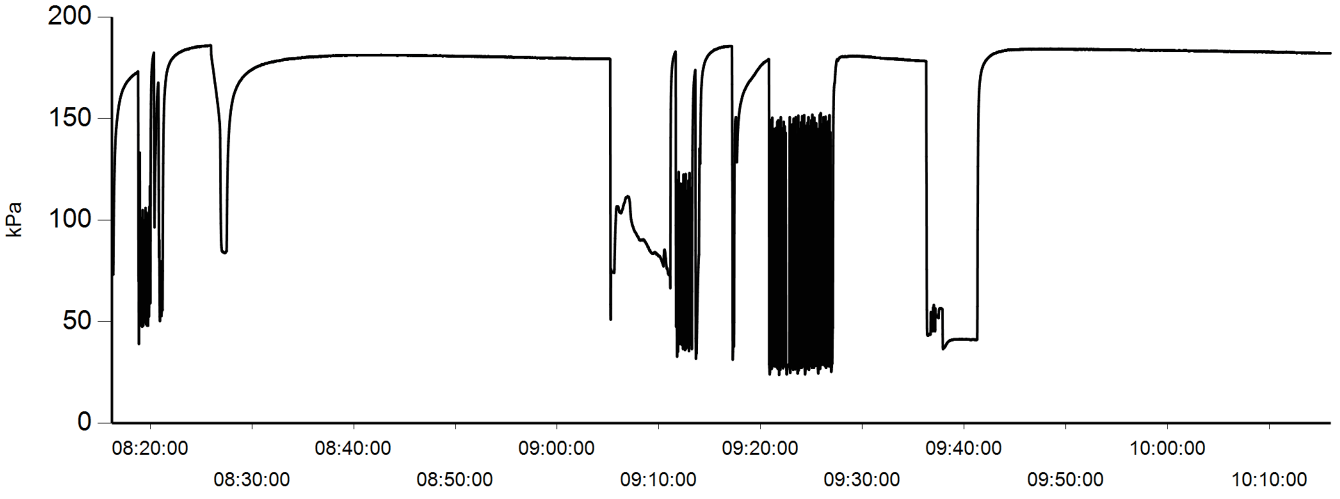

Synthera+ HPLC (HP183703) - Synthesis evolution of Compressed air

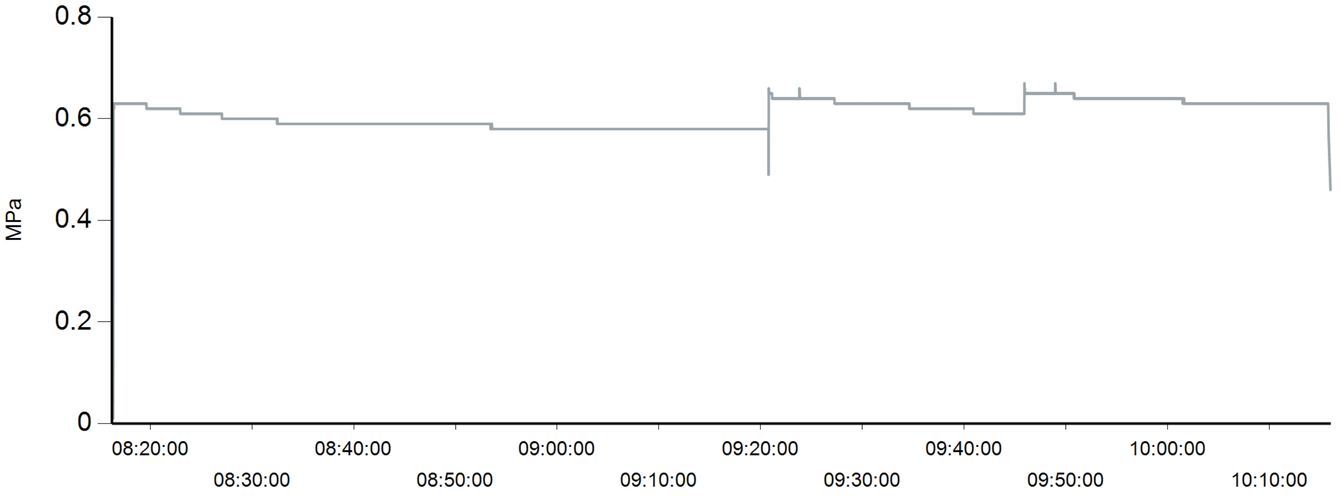

Synthesis report

SW: Version 1.1 | October 2018

Document id: S2005270363 Batch Nr: PE2I-200527-2

Synthera+ HPLC (HP183703) - Synthesis evolution of Aux. Signal

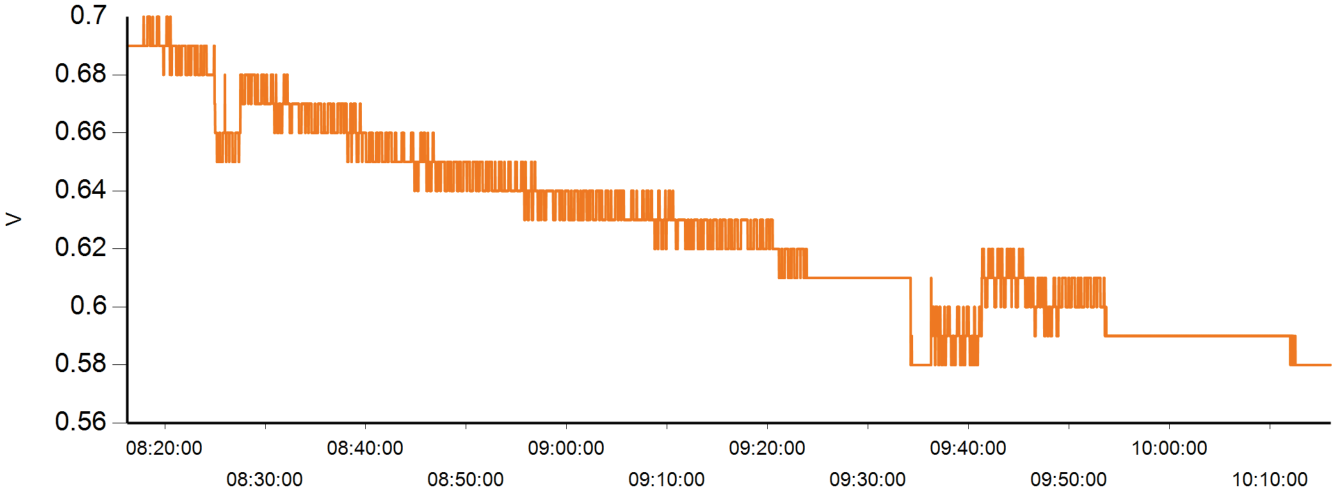

Synthera+ HPLC (HP183703) - Synthesis evolution of UV detector

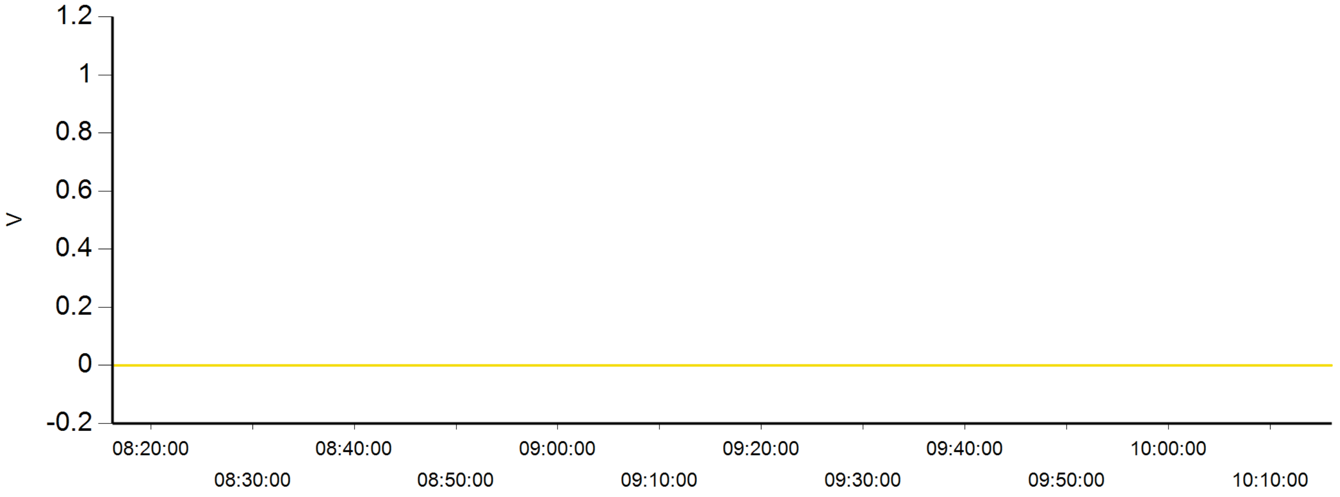

Synthera+ HPLC (HP183703) - Synthesis evolution of HPLC Pump

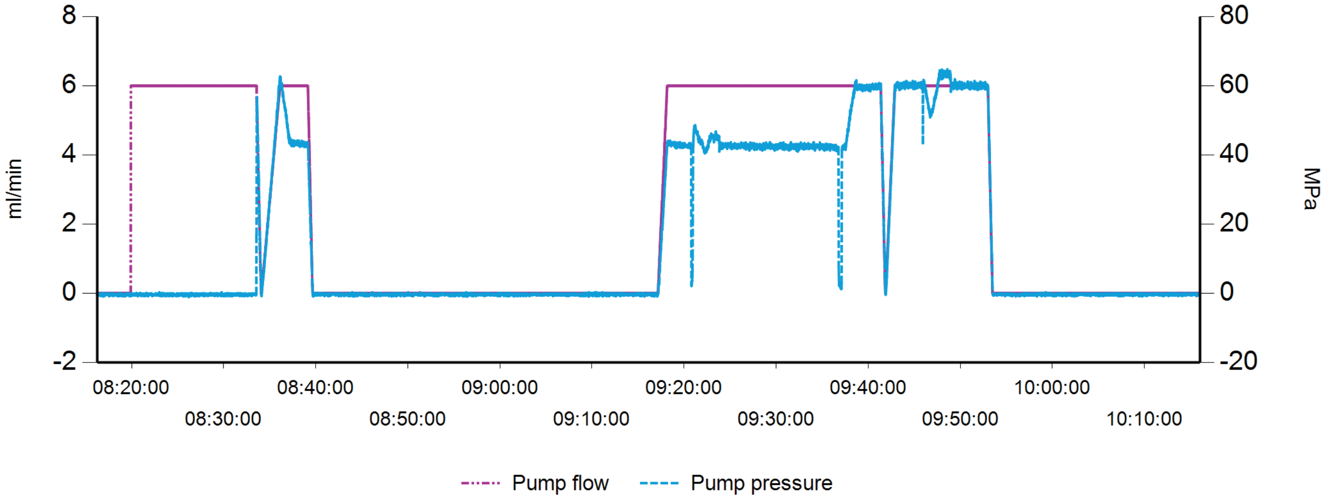

Synthesis report

SW: Version 1.1 | October 2018

Document id: S2008180422 Batch Nr: Vasko drying DMSO3

Start at 8/18/2020 11:17:13 AM
End at 8/18/2020 11:33:40 AM
Duration: 0:16:26

User: Denholdt Charlotte (CLD)
Level: Supervisor

[18F]Fluorine
FE-PE2I
Status: Synthesis completed with skipped event

Start activity:
End activity:
Estimated yield: % n.d.c.

Recipe: FE-PE2I (v4)
Script: FE-PE2I (v17)

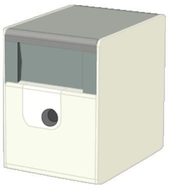

Synthera+
IBA SN: SM182802
Site Id: Synthera+ #1
HW: 1246398

IFP: 2-2560-0618201547253
Status: Single use IFP found
Consumable:
NA

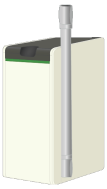

Synthera+ HPLC
IBA SN: HP183703
Site Id: HPLC #1
HW: 1250402

Comments:

Author	Manager	Quality
Name:	Name:	Name:
Date:	Date:	Date:
Comment:	Comment:	Comment:

Synthesis report

Document id: S2008180422 Batch Nr: Vasko drying DMSO3

Synthesis report

SW: Version 1.1 | October 2018

Document id: S2008180422 Batch Nr: Vasko drying DMSO3

Synthera+ (SM182802) - Details

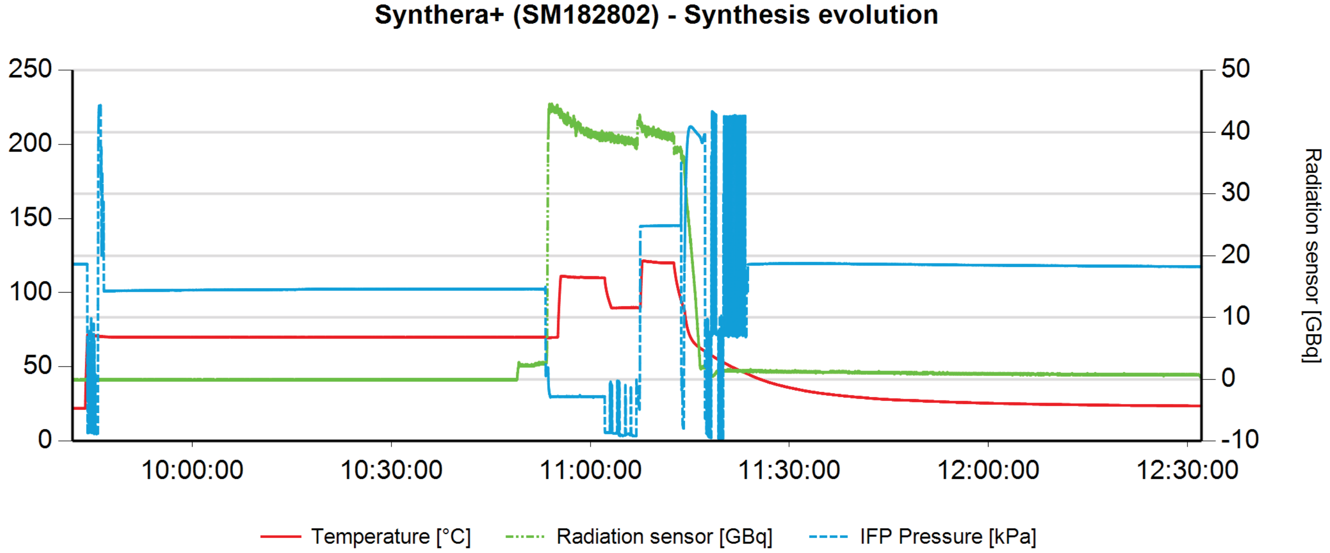

11:23:56 AM	Final sequence status: Completed		
9:41:56 AM	*Command: Load*		
9:43:15 AM	*Command: Start*		

	Sequence evolution	
9:43:27 AM	**Initialization**	**Completed**
9:43:50 AM	**Valve rotation test**	**Completed**
9:44:07 AM	**Temperature test and pre-heat**	**Completed**
9:44:06 AM	*Action: Signal report*	Temperature value: 64,03 °C
9:45:24 AM	**Purging of the IFP**	**Completed**
9:45:43 AM	**Vacuum test**	**Completed**
9:45:30 AM	*Action: Signal report*	IFP Pressure value: 4,92 kPa
9:45:43 AM	*Action: Signal report*	IFP Pressure value: 5,08 kPa
9:46:08 AM	**Pressure test**	**Completed**
9:45:55 AM	*Action: Signal report*	IFP Pressure value: 224,38 kPa
9:46:07 AM	*Action: Signal report*	IFP Pressure value: 225,71 kPa
10:12:03 AM	**Test tube 1 - 2 - 3 - 4**	**Completed**
10:51:04 AM	**Ready to start**	**Completed**
10:51:03 AM	*Action: Resume (WD;USR;;)*	
10:53:10 AM	**Trapping Fluorine-18**	**Completed**
10:55:06 AM	**Elution Fluorine-18**	**Completed**
10:55:05 AM	*Action: Signal report*	Radiation sensor value: 43,43 GBq
11:07:03 AM	**Drying Fluorine-18**	**Completed**
11:06:21 AM	*Action: Signal report*	IFP Pressure value: 3,75 kPa
11:07:31 AM	**Precursor to reactor**	**Completed**

Synthesis report

Document id: S2008180422 Batch Nr: Vasko drying DMSO3

11:13:36 AM	**Labelling**	**Completed**
11:14:08 AM	**Buffer to reactor**	**Completed**
11:17:13 AM	**Transfer to HPLC**	**Completed**
11:23:43 AM	**Purging of the pump**	**Completed**
11:23:55 AM	**Re-initialization**	**Completed**

Synthesis report

Document id: S2008180422 Batch Nr: Vasko drying DMSO3

Synthera+ HPLC (HP183703) - Details

Synthera+ HPLC (HP183703) - Synthesis evolution of Radiation sensor

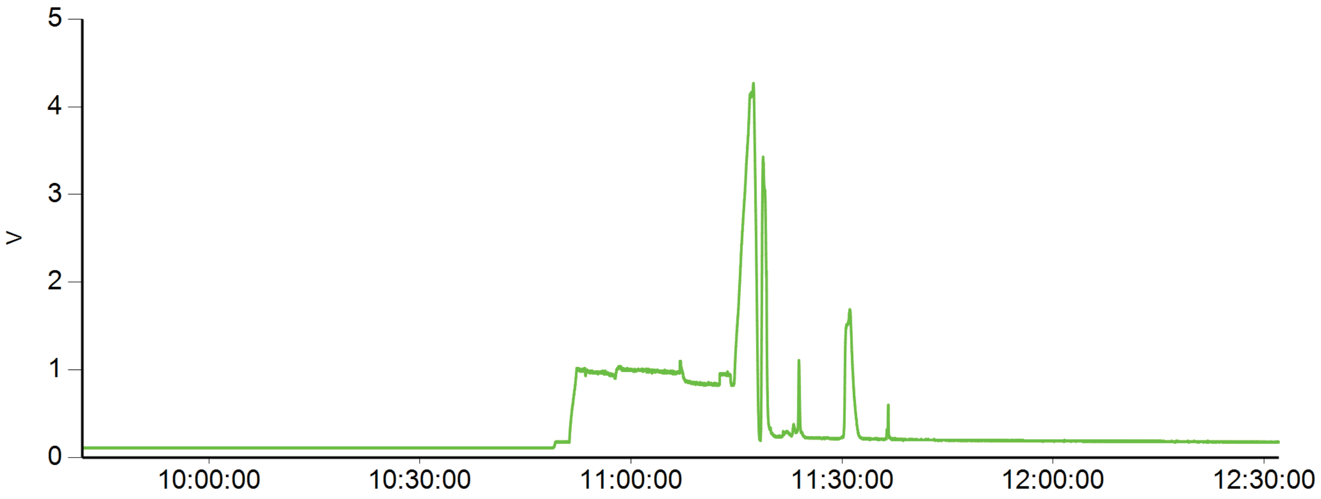

12:01:52 PM	Final sequence status: Completed with skipped event		
9:41:56 AM	*Command: Load*		
9:43:15 AM	*Command: Start*		

	<u>Sequence evolution</u>	
9:59:28 AM	**Pre-cleaning**	**Completed**
10:12:03 AM	**Conditioning**	**Completed**
11:14:39 AM	**Ready to start**	**Completed**
11:17:13 AM	**Wait transfer from Synthera+**	**Completed**
11:33:41 AM	**Purification - Collection**	Completed with skipped event
11:30:20 AM	*Action: Resume (WD;V02;1;)*	
11:32:03 AM	*Action: Resume (WD;V02;0;)*	
11:33:39 AM	*Action: Skip (WA;TIM;300;)*	
12:01:51 PM	**Cleaning**	**Completed**

Synthesis report

SW: Version 1.1 | October 2018

Document id: S2008180422 Batch Nr: Vasko drying DMSO3

Synthera+ (SM182802) - Synthesis evolution of Temperature

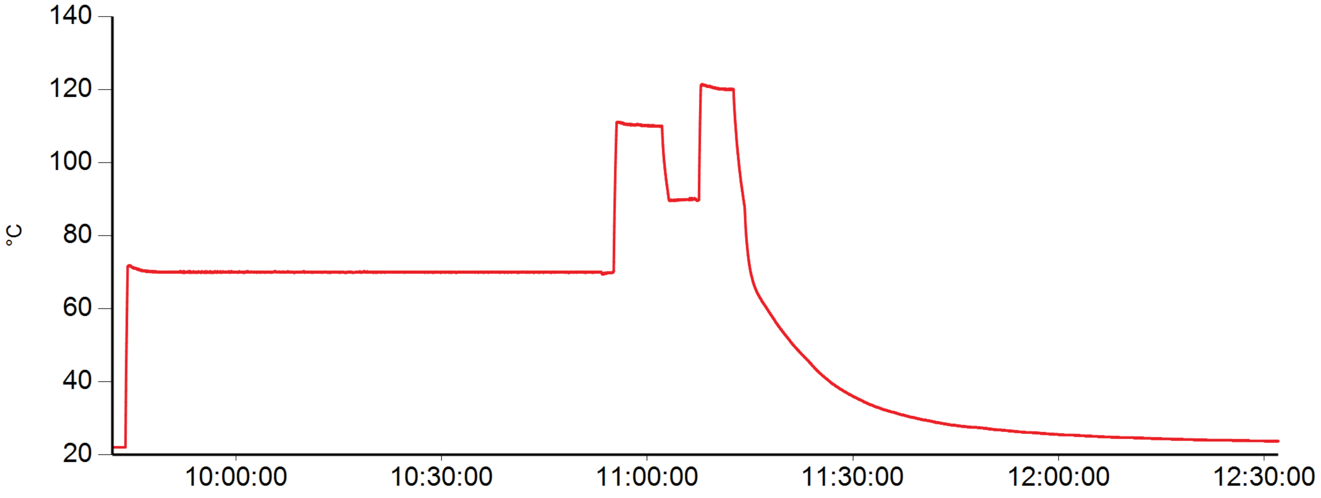

Synthera+ (SM182802) - Synthesis evolution of Radiation sensor

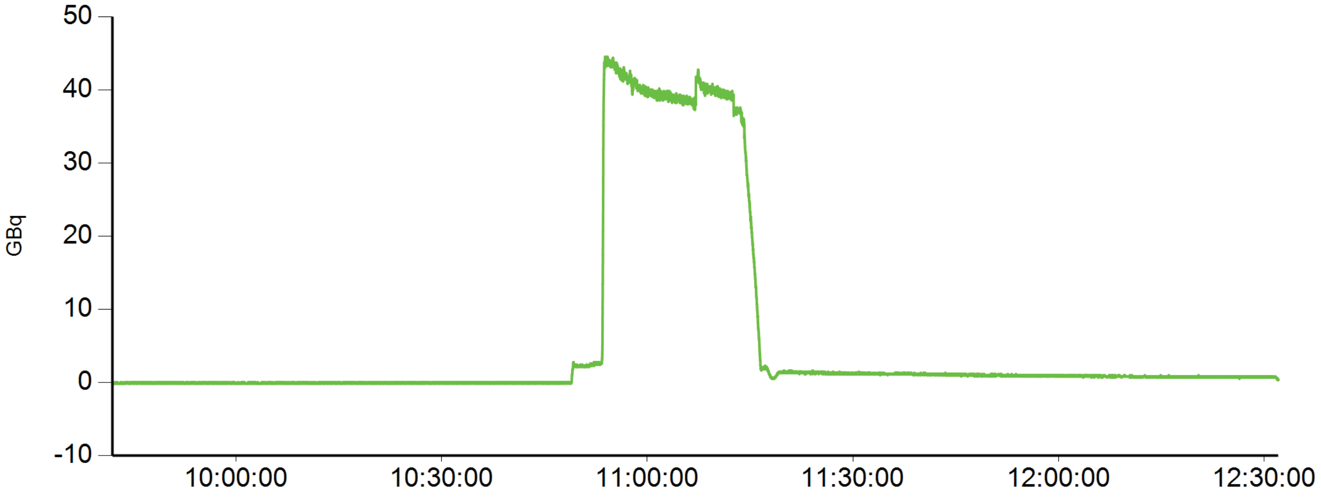

Synthera+ (SM182802) - Synthesis evolution of Compressed air

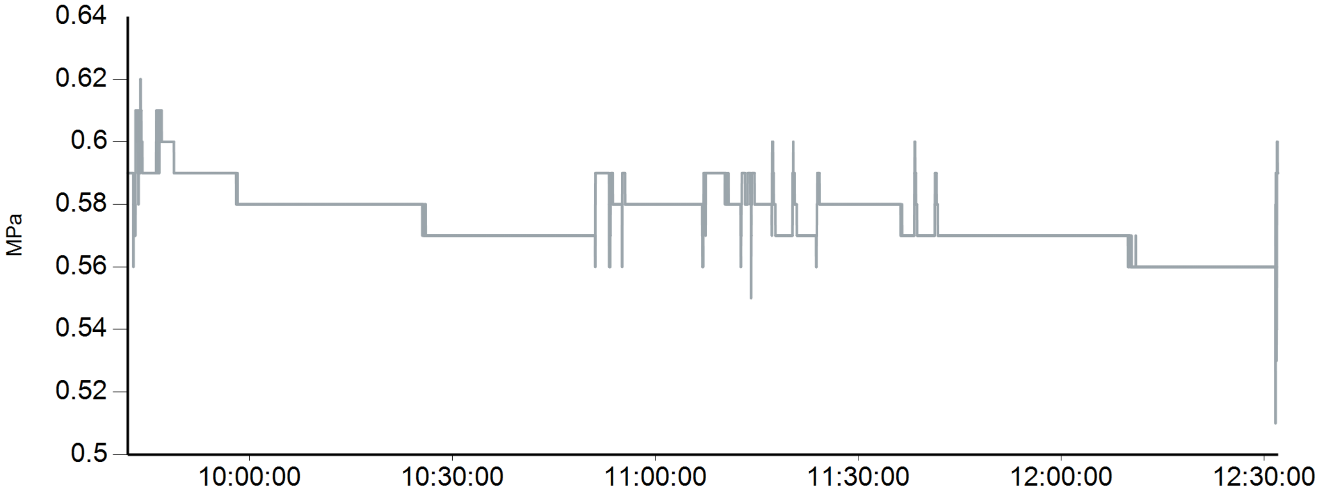

Synthesis report

Document id: S2008180422 Batch Nr: Vasko drying DMSO3

Synthera+ (SM182802) - Synthesis evolution of IFP Pressure

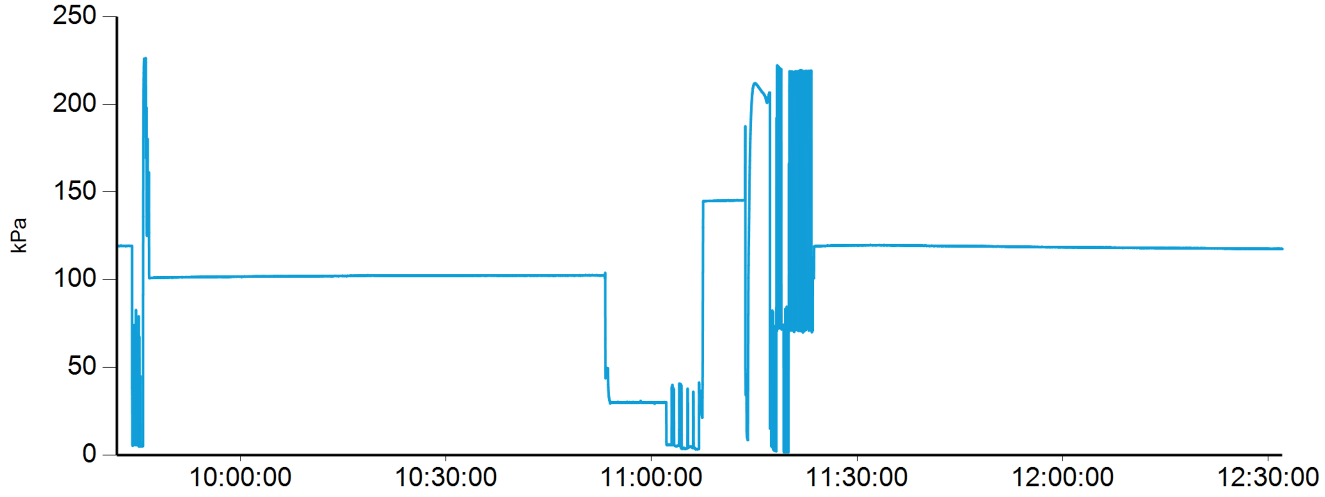

Synthera+ (SM182802) - Synthesis evolution of Aux. Signal

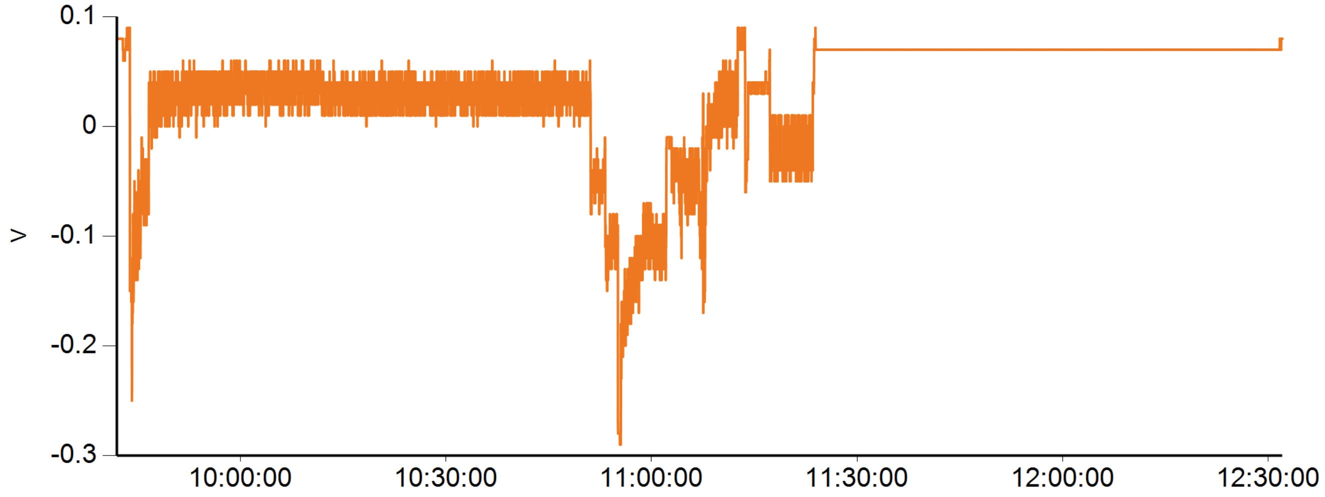

Synthesis report

SW: Version 1.1 | October 2018

Document id: S2008180422 Batch Nr: Vasko drying DMSO3

Synthera+ HPLC (HP183703) - Synthesis evolution of Radiation sensor

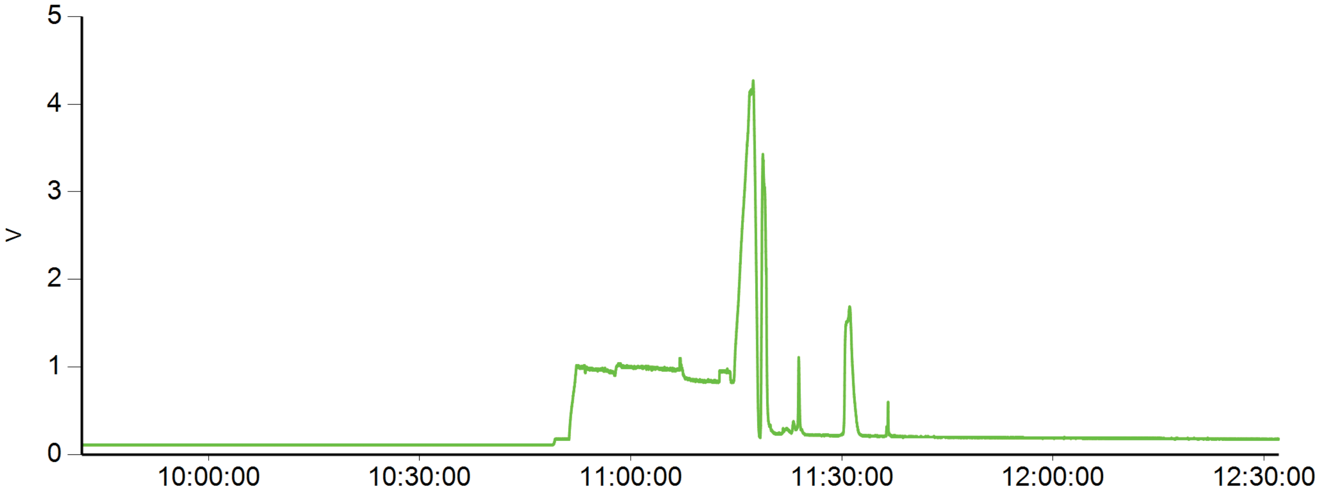

Synthera+ HPLC (HP183703) - Synthesis evolution of Inert gas pressure

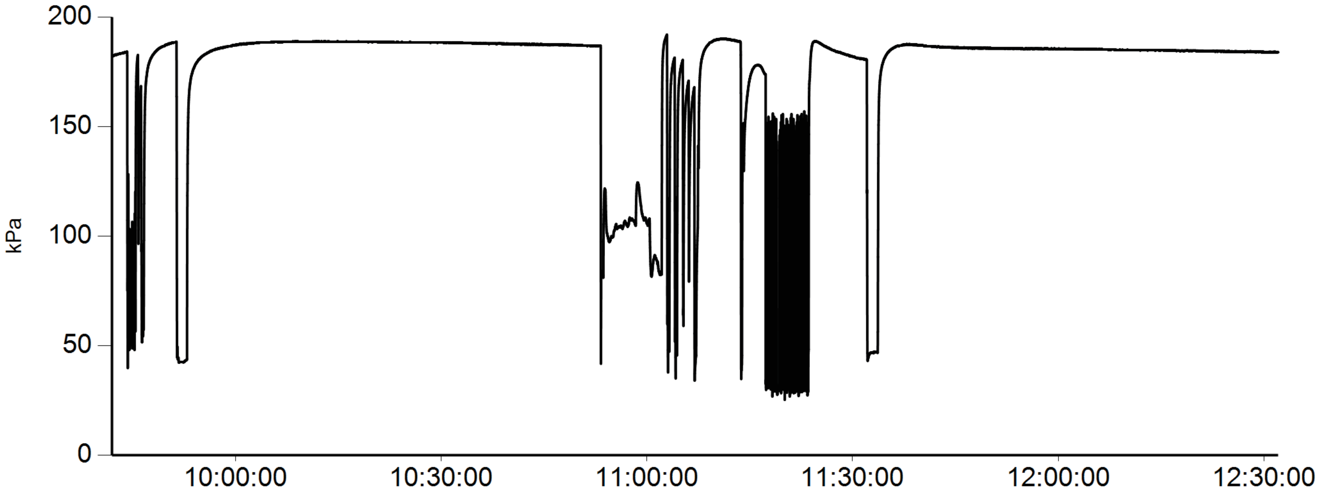

Synthera+ HPLC (HP183703) - Synthesis evolution of Compressed air

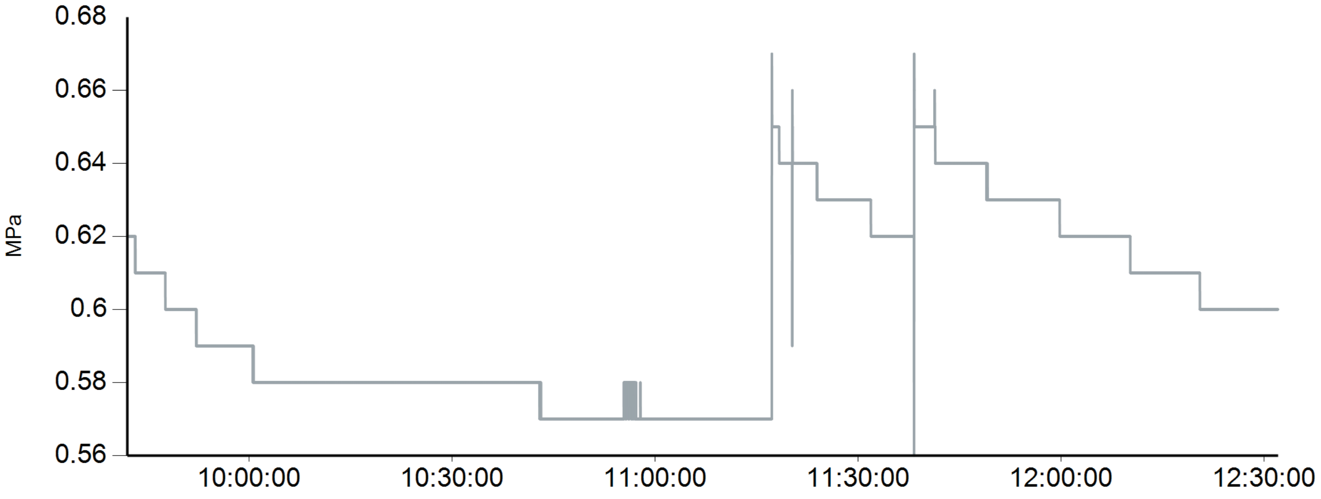

Synthesis report

Document id: S2008180422 Batch Nr: Vasko drying DMSO3

Synthera+ HPLC (HP183703) - Synthesis evolution of Aux. Signal

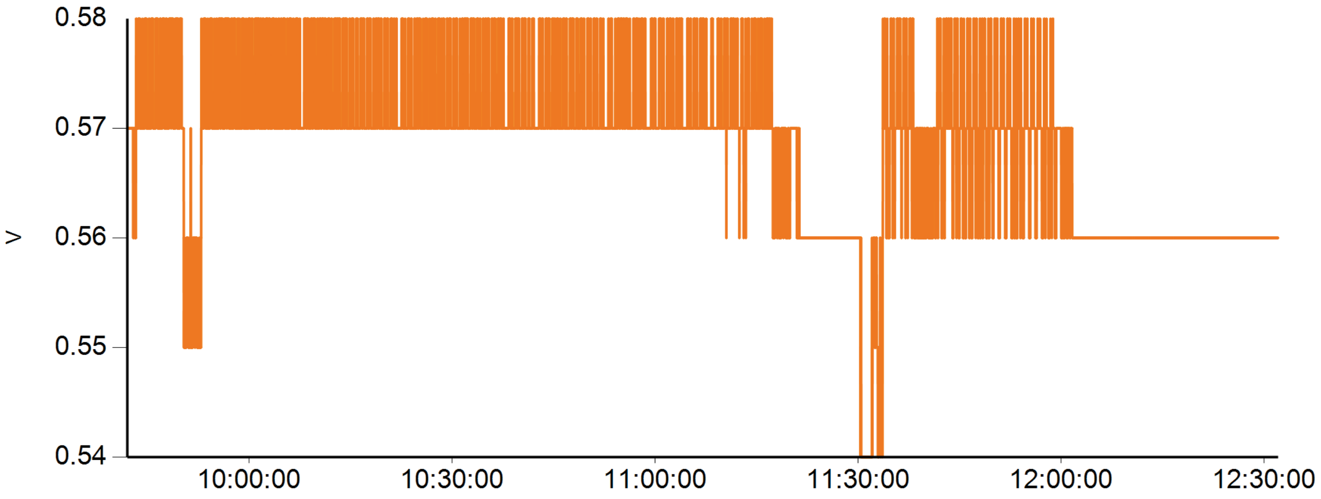

Synthera+ HPLC (HP183703) - Synthesis evolution of UV detector

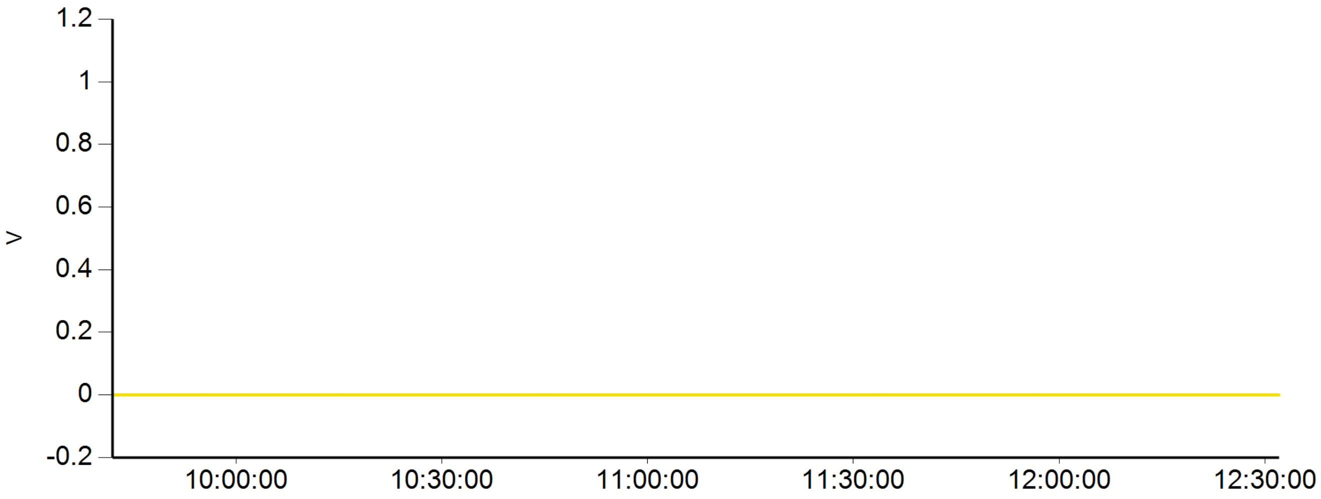

Synthera+ HPLC (HP183703) - Synthesis evolution of HPLC Pump

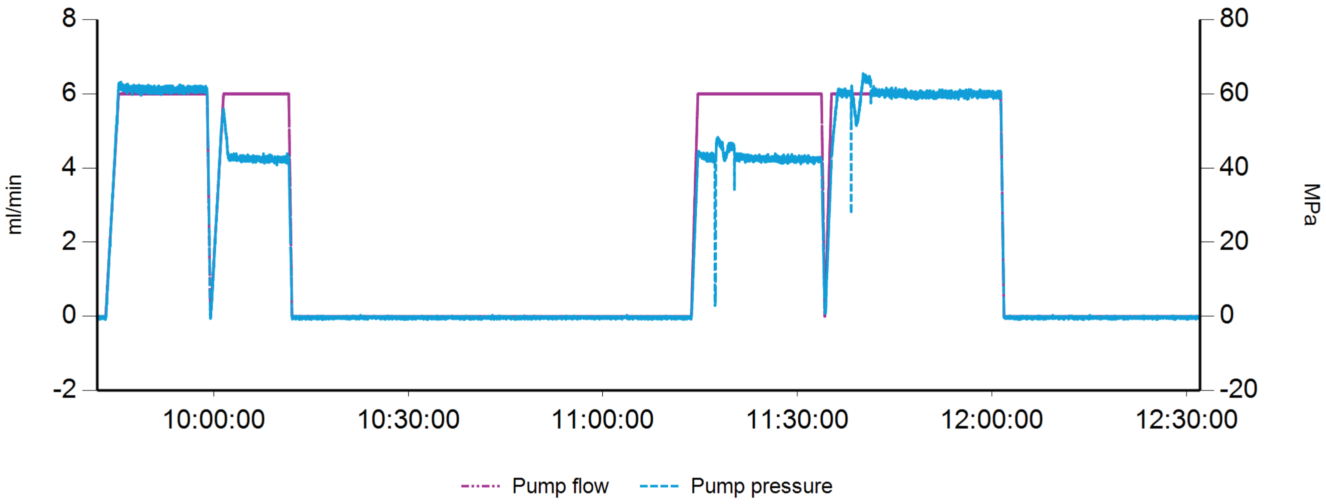

Supplement: Supplementary file 1 [file pharmaceuticals-14-00601-s001.zip › report.pdf]
